# Global and regional gastric cancer burden and age-related survival disparities: complementary analyses of GBD 2021 and SEER data

**DOI:** 10.3389/fonc.2026.1880227

**Published:** 2026-10-20

**Authors:** Jie Cheng, Ruitong Sun, Yun Gong, Die Fan, Lixing Wang

**Affiliations:** 1 Department of Gastrointestinal Surgery, The Second Affiliated Hospital of Kunming Medical University, Kunming, China; 2 Faculty of Life Science and Technology, Kunming University of Science and Technology, Kunming, China; 3 Pediatrics Program, 2023 Cohort, The First Clinical Medical College, Kunming Medical University, Kunming, China; 4 Peking University Cancer Hospital Department of Gynecologic Oncology, Yunnan Cancer Hospital, The Third Affiliated Hospital of Kunming Medical University, Kunming, China

**Keywords:** age-related disparities, age-standardized rates, gastric cancer, global burden of disease, SEER, survival analysis

## Abstract

**Background:**

Global gastric cancer burden and patient-level survival disparities are often examined separately. This separation limits understanding of how population-level age patterns relate to clinical presentation and prognosis. We used GBD 2021 and SEER as complementary evidence sources to evaluate global and regional burden and age-related survival disparities in gastric cancer.

**Methods:**

GBD 2021 estimates assessed incidence, deaths, and DALYs during 1990–2021 at Global, five SDI groups, and GBD-region levels. Numbers, age-standardized rates, 95% uncertainty intervals (UIs), and estimated annual percentage changes (EAPCs) were reported. An adenocarcinoma-focused SEER cohort diagnosed in 2000–2021 was evaluated across AYA (15–39 years), middle-aged (40–64 years), and older (>=65 years) groups using overall survival (OS), conventional cancer-specific survival, cumulative-incidence functions (CIFs), Gray tests, and cause-specific Cox models.

**Results:**

Global incident cases increased from 980,899 (95% UI 891,307-1,072,236) in 1990 to 1,230,233 (1,052,350-1,409,970) in 2021, whereas the age-standardized incidence rate declined from 24.76 to 14.33 per 100,000. The SEER cohort included 89,798 patients; OS and cause-specific cohorts included 89,622 and 88,109 patients. Compared with AYA, follow-up-averaged primary Model 2 associations for older adults were HR 1.73 (95% CI 1.66-1.79) for all-cause death and 1.52 (1.46-1.58) for gastric-cancer death. CIFs showed greater non-cancer mortality among older adults.

**Conclusions:**

Gastric cancer remains a substantial population burden despite declining age-standardized rates. Complementary population-level and U.S. patient-level evidence indicates age-related heterogeneity in burden, clinical presentation, competing mortality, and survival associations. These observations support population-level burden reduction alongside age-aware clinical management.

## 1 Introduction

Gastric cancer remains a major contributor to cancer morbidity and mortality worldwide (1–3). Improvements in prevention, diagnosis, and treatment have reduced age-standardized rates in many settings, yet the absolute number of patients requiring diagnosis, treatment, and long-term care remains large (2, 3). For health systems, this distinction matters: age-standardized rates describe underlying risk after accounting for population structure, whereas all-age numbers reflect the actual service burden that must be managed (4).

Overall burden estimates alone do not capture how gastric cancer affects different age groups. Gastric cancer is strongly age dependent, but younger patients and older adults raise different clinical questions (5–7). AYA patients may present with distinctive histology, stage distribution, genetic susceptibility, diagnostic delay, and survivorship needs (5–9). Older adults, in contrast, often face competing mortality, frailty, comorbidity, and treatment-tolerance issues (10–12). A framework that includes age-specific burden and age-related outcomes is therefore more informative than a single population-average estimate.

GBD and SEER answer different parts of this problem. GBD 2021 provides modeled estimates of incidence, deaths, DALYs, and temporal trends across global and regional strata, allowing comparison by SDI and GBD region (4, 13). SEER provides patient-level registry data with tumor characteristics, treatment variables, and survival outcomes in U.S. registry areas (14, 15). Used alone, GBD cannot describe individual-level clinicopathological patterns or survival after diagnosis, while SEER cannot represent the global distribution of disease burden.

Previous studies have commonly focused either on global or regional burden trends or on patient-level prognostic factors (2, 3, 5, 13). Fewer analyses place these two forms of evidence in the same interpretive frame, especially around age. Such an approach does not merge the data sources or imply causation between global trends and individual outcomes; rather, it asks how population-level age patterns and patient-level age disparities can jointly inform gastric cancer prevention and management.

In this study, we analyzed GBD 2021 stomach cancer estimates for Global, SDI groups, and GBD regional burden from 1990–2021 and used an adenocarcinoma-focused SEER cohort to evaluate age-related clinicopathological differences and survival. Our aim was to describe how gastric cancer burden varies across populations and ages, and how age relates to clinical presentation, competing mortality, and survival at the patient level.

## 2 Methods

### 2.1 Study design

This was a secondary analysis of two public data sources. GBD 2021 estimates described population-level gastric cancer burden (4), whereas SEER registry data characterized U.S. patient-level age-related clinicopathological and survival patterns (14, 15). The data sources were analyzed separately and interpreted complementarily; no individual-level linkage, pooled model, or global extrapolation from SEER was performed.

### 2.2 GBD 2021 data source and extraction

GBD 2021 stomach-cancer estimates were obtained from the GBD Results Tool for Global, five SDI groups (High, High-middle, Middle, Low-middle, and Low SDI), and native GBD regions (4). Country-level data were not included; national rankings, country-level EAPCs, and maps were not undertaken. The source fields val, lower, and upper represented point estimates and 95% UIs.

Incidence, deaths, and disability-adjusted life-years (DALYs) were assessed as numbers and rates for 1990-2021, both sexes and sex-specific strata, all ages, age-standardized estimates, and 5-year age groups. AYA was 15–39 years, middle-aged adults 40–64 years, and older adults >=65 years. Numbers were summed across constituent 5-year groups; rates were retained at native 5-year ages and were not aggregated without population weights.

For GBD numbers, estimates from constituent 5-year age groups were summed to obtain the three broader age groups. Rates were not summed or averaged across age groups because population weights were not available. Age-specific rates were therefore retained at the original 5-year age-group level, and EAPC analyses were restricted to rates, mainly age-standardized rates.

### 2.3 GBD trend and EAPC analyses

Global trends were summarized using all-age numbers and age-standardized rates. EAPCs were estimated for positive rates by log-linear regression of ln(rate) on calendar year; their regression-derived 95% CIs are distinct from GBD 95% UIs (16).

### 2.4 SEER data source and cohort selection

The SEER component used patient-level SEER*Stat case-listing data from Incidence - SEER Research Data, 17 Registries, Nov 2025 Sub (2000–2023), linked to county attributes (14, 15). The original SEER*Stat source cohort was restricted to stomach primary site C16.x, malignant behavior, diagnosis during 2000-2021, age >=15 years, known age, male or female sex, and one primary tumor only or the first of two or more primaries; its prespecified morphology source range was 8140–8239 or 8250-8579. After morphology refinement according to SEER Rare Cancer Classification 8.1, the final adenocarcinoma-focused cohort retained 36 observed eligible morphology codes and excluded 29 records with 11 observed noneligible codes. Eligibility, exclusions, and the retained morphology mapping are detailed in **Figure 1**; **Supplementary Table S1**. The GBD stomach-cancer and SEER adenocarcinoma-focused definitions are not identical.

**Figure 1:**
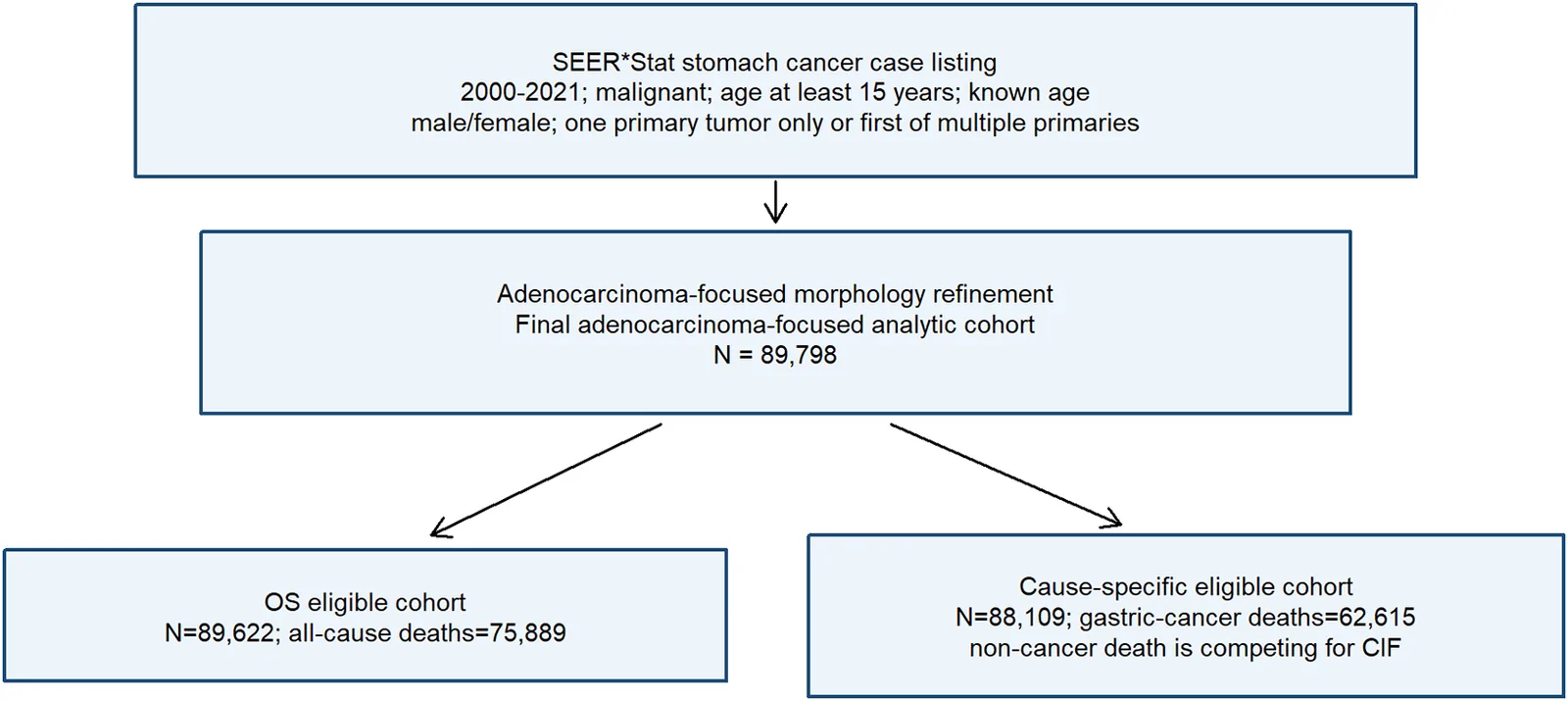
SEER adenocarcinoma-focused cohort-selection flowchart. The diagram shows the final analytic cohort and endpoint-eligible cohorts. Detailed ICD-O morphology eligibility is provided in **Supplementary Table S1**.

Variables included sex, race/ethnicity, diagnosis period, primary site, histologic subtype, grade, stage, and registry-coded treatment variables. Chemotherapy No/Unknown was not interpreted as confirmed absence of therapy.

### 2.5 SEER survival outcomes

Survival time was observed Survival Days divided by 365.25. Exact zero-day observations remained at time=0 in primary Kaplan-Meier, Cox, and CIF analyses; no positive offset was applied. OS used all-cause death. Conventional CSS treated gastric-cancer-attributable death as the event and censored non-cancer death. CIFs estimated gastric-cancer and non-cancer death; Gray tests compared CIFs. Cause-specific Cox models treated non-cancer death as censoring for the cause-specific hazard (17). A sensitivity analysis excluded exact-zero records.

### 2.6 Statistical analysis

Categorical baseline variables were compared by chi-square tests and summarized with maximum absolute pairwise standardized mean differences (SMDs) (18). Reverse Kaplan-Meier estimated follow-up (19). OS and conventional CSS used Kaplan-Meier/log-rank methods (20). Model 1 included demographic factors and diagnosis period. Primary clinicopathologic-adjusted Model 2 additionally included primary site, histologic subtype, grade, and stage. Model 3 additionally included registry-recorded treatment variables and was exploratory because treatment timing, indication, intent, and key confounders were incompletely captured (21). Statistical analyses were conducted in R version 4.5.1; survival analyses, Cox models, and proportional-hazards diagnostics used survival version 3.8-3, and CIFs and Gray tests used cmprsk version 2.2-12. GBD processing and EAPC calculations were conducted in Python 3.13.9 using pandas 2.3.3.

Formal proportional-hazards diagnostics were assessed (22). Because age violated proportional hazards, fixed primary Model 2 age HRs are reported as follow-up-averaged Cox associations under a proportional-effects approximation; secondary piecewise estimates are interval-averaged associations. Older subgroups (65-74, 75-84, and >=85 years) were descriptive only.

## 3 Results

### 3.1 Global burden and temporal trends

GBD 2021 showed that the global absolute burden of gastric cancer remained substantial from 1990-2021, even as age-standardized rates declined (**Figure 2**; **Table 1**). Incident cases increased from 980,899 (95% UI 891,307-1,072,236) to 1,230,233 (1,052,350-1,409,970), and deaths from 854,185 (772,885-939,973) to 954,374 (821,751-1,089,577). DALYs decreased from 23.24 million (20.61-25.53) to 22.79 million (19.58-26.12).

**Figure 2:**
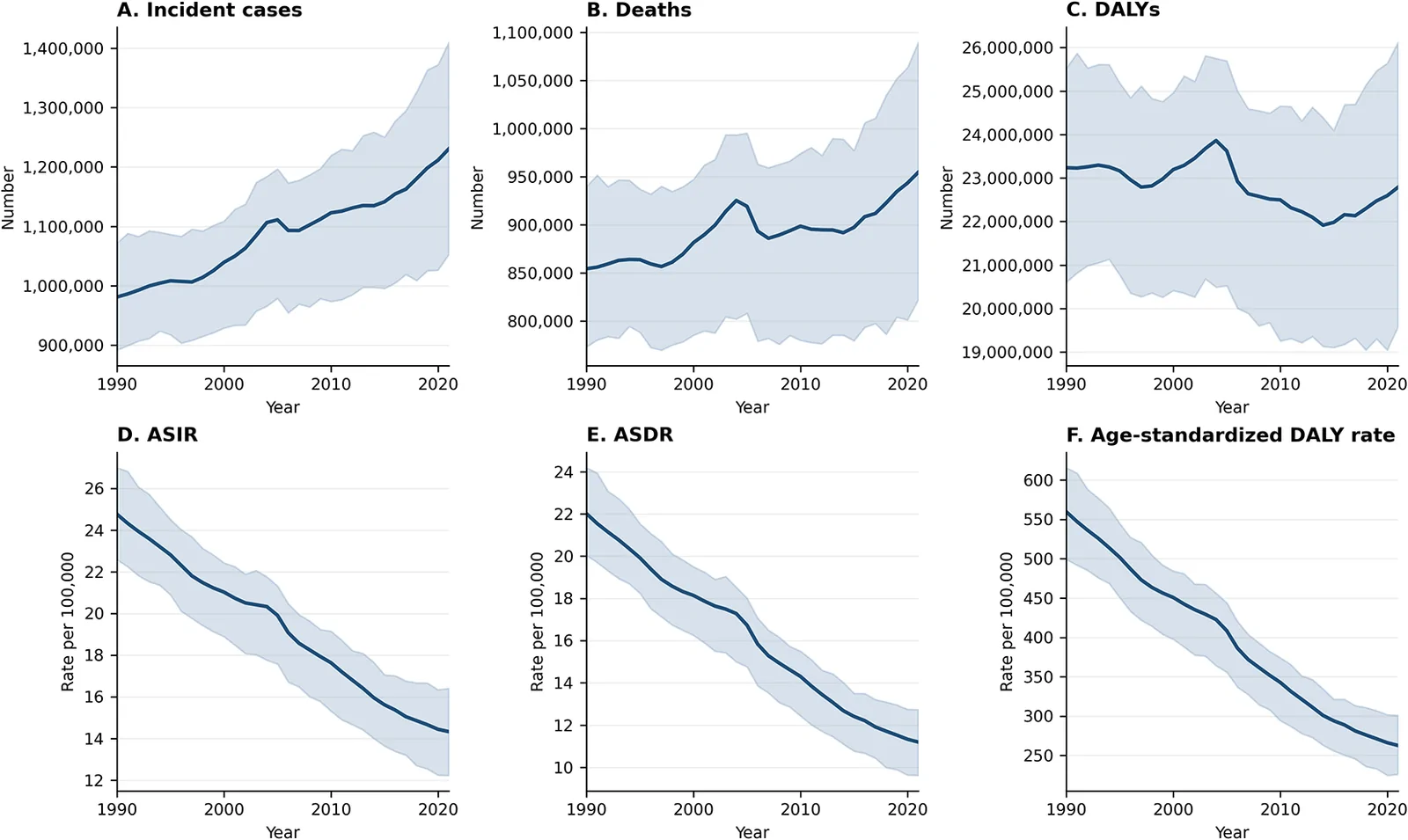
Global stomach-cancer burden, 1990-2021. **(A–C)** show all-age numbers for incidence, deaths, and DALYs; **(D–F)** show age-standardized rates. Shaded bands represent annual 95% uncertainty intervals (UIs).

**Table 1:** GBD 2021 global stomach-cancer burden in 1990 and 2021.

Measure	Metric	1990 estimate (95% UI)	2021 estimate (95% UI)	Absolute change	Percent change	EAPC (95% CI)
Incidence	Number	980,899 (891,307-1,072,236)	1,230,233 (1,052,350-1,409,970)	249,333	25.4%	Not applicable
Incidence	Rate	24.76 (22.58-27.00)	14.33 (12.23-16.41)	-10.44	-42.1%	-1.81% (-1.87% to -1.75%)
Deaths	Number	854,185 (772,885-939,973)	954,374 (821,751-1,089,577)	100,189	11.7%	Not applicable
Deaths	Rate	22.01 (20.03-24.19)	11.20 (9.62-12.73)	-10.81	-49.1%	-2.26% (-2.34% to -2.18%)
DALYs	Number	23,237,292 (20,605,349-25,526,194)	22,786,633 (19,576,344-26,118,869)	-450,659	-1.9%	Not applicable
DALYs	Rate	559.72 (499.09-615.77)	262.75 (226.08-301.02)	-296.97	-53.1%	-2.54% (-2.62% to -2.46%)

Values are GBD point estimates with 95% UIs; rates are per 100,000 population. EAPC 95% CIs are regression-derived and distinct from source UIs. DALYs, disability-adjusted life-years; EAPC, estimated annual percentage change.

ASIR declined from 24.76 to 14.33 per 100,000, ASDR from 22.01 to 11.20, and the age-standardized DALY rate from 559.72 to 262.75. EAPCs were -1.81% (95% CI -1.87% to -1.75%) for ASIR, -2.26% (-2.34% to -2.18%) for ASDR, and -2.54% (-2.62% to -2.46%) for the age-standardized DALY rate. Shaded bands in **Figure 2** show annual GBD 95% UIs.

### 3.2 Age- and sex-specific burden

Age-specific GBD patterns provided the population-level context for the SEER age-group analysis (**Figure 3**). The figure compares 1990 and 2021 age-specific rates for both sexes and sex-specific strata. Rates rose steeply with age and were generally higher among males; these population patterns do not link or imply causation for SEER survival results.

**Figure 3:**
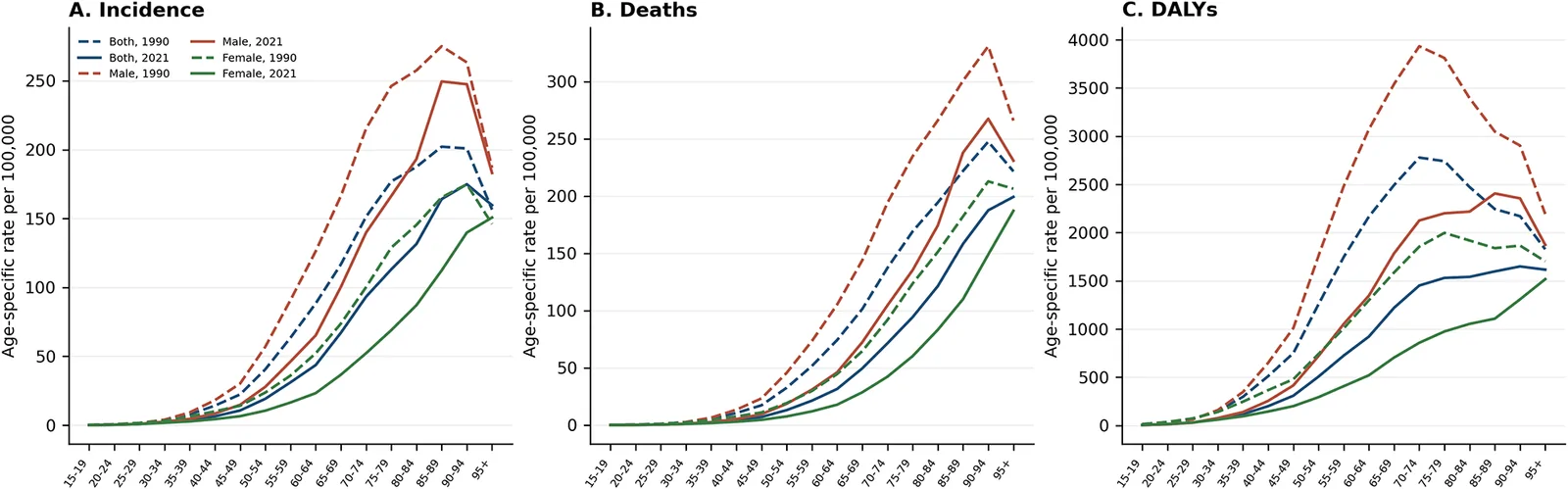
Age- and sex-specific stomach-cancer burden in 1990 and 2021. **(A)** Incidence, **(B)** deaths, and **(C)** DALYs. Each panel shows age-specific rates for both sexes, males, and females in 1990 and 2021. The figure was regenerated from validated GBD 2021 data through scripted analysis.

Among both sexes, AYA incident cases decreased from 53,639 in 1990 to 42,039 in 2021 (-21.6%), and middle-aged adult incident cases were relatively stable, changing from 438,730 to 431,536 (-1.6%). In contrast, incident cases among older adults increased from 488,530 to 756,658 (+54.9%). A similar age shift was observed for deaths: AYA deaths decreased from 38,152 to 24,272 (-36.4%), middle-aged adult deaths decreased from 355,149 to 298,709 (-15.9%), whereas older-adult deaths increased from 460,883 to 631,393 (+37.0%). These age patterns provided population-level context for patient-level comparisons across AYA, middle-aged adults, and older adults in SEER.

### 3.3 SDI and regional burden heterogeneity

The burden of gastric cancer differed substantially across SDI groups and GBD regions. In 2021, ASIR was highest in High-middle SDI (19.62 per 100,000) and lowest in Low-middle SDI (7.68 per 100,000). ASDR was highest in High-middle SDI (14.93 per 100,000) and lowest in High SDI (6.83 per 100,000). The age-standardized DALY rate was also highest in High-middle SDI (353.18 per 100,000) and lowest in High SDI (146.10 per 100,000). Detailed SDI-specific EAPC estimates are provided in **Supplementary Table S2** and **Supplementary Figure S1**.

Across GBD regions, East Asia had the highest 2021 ASIR (28.64 per 100,000), Andean Latin America had the highest ASDR (21.33 per 100,000), and East Asia had the highest age-standardized DALY rate (496.94 per 100,000). GBD region-level burden and EAPC estimates are shown in **Supplementary Table S3** and **Supplementary Figure S2**. Detailed age-specific estimates are provided in **Supplementary Table S4**.

### 3.4 SEER cohort and baseline characteristics

After adenocarcinoma-focused morphology refinement, the final SEER cohort included 89,798 patients (**Figure 1**): 3,588 AYA (4.0%), 33,643 middle-aged adults (37.5%), and 52,567 older adults (58.5%). Baseline distributions differed across age groups (**Table 2**); maximum pairwise SMDs were largest for chemotherapy (0.770), histology (0.631), race/ethnicity (0.628), and summary stage (0.533). Full baseline data are in **Supplementary Table S5**.

**Table 2:** SEER baseline characteristics by age group.

Variable	Category	AYA	Middle-aged adults	Older adults	Max absolute SMD	P value
Total	N	3588	33643	52567		
Sex	Female	1673 (46.6%)	11106 (33.0%)	20559 (39.1%)	0.281	P<0.001
Sex	Male	1915 (53.4%)	22537 (67.0%)	32008 (60.9%)		P<0.001
Race/ethnicity	Hispanic	1594 (44.4%)	8452 (25.1%)	8830 (16.8%)	0.628	P<0.001
Race/ethnicity	Non-Hispanic Asian or Pacific Islander	572 (15.9%)	4857 (14.4%)	8892 (16.9%)		P<0.001
Race/ethnicity	Non-Hispanic Black	408 (11.4%)	4527 (13.5%)	5920 (11.3%)		P<0.001
Race/ethnicity	Non-Hispanic White	938 (26.1%)	15331 (45.6%)	28381 (54.0%)		P<0.001
Race/ethnicity	Other/Unknown	76 (2.1%)	476 (1.4%)	544 (1.0%)		P<0.001
Diagnosis period	2000–2005	940 (26.2%)	8411 (25.0%)	15046 (28.6%)	0.082	P<0.001
Diagnosis period	2006–2011	908 (25.3%)	9075 (27.0%)	13989 (26.6%)		P<0.001
Diagnosis period	2012–2017	1035 (28.8%)	9934 (29.5%)	14121 (26.9%)		P<0.001
Diagnosis period	2018–2021	705 (19.6%)	6223 (18.5%)	9411 (17.9%)		P<0.001
Primary site	Cardia	755 (21.0%)	11531 (34.3%)	15454 (29.4%)	0.299	P<0.001
Primary site	Non-cardia	1674 (46.7%)	14399 (42.8%)	25826 (49.1%)		P<0.001
Primary site	Stomach NOS/other	1159 (32.3%)	7713 (22.9%)	11287 (21.5%)		P<0.001
Histology	Adenocarcinoma, NOS/common variants	2001 (55.8%)	24116 (71.7%)	43259 (82.3%)	0.631	P<0.001
Histology	Mucinous adenocarcinoma	65 (1.8%)	687 (2.0%)	1180 (2.2%)		P<0.001
Histology	Other retained rare adenocarcinoma variant	14 (0.4%)	211 (0.6%)	316 (0.6%)		P<0.001
Histology	Signet-ring cell carcinoma	1508 (42.0%)	8629 (25.6%)	7812 (14.9%)		P<0.001
Grade	Poorly/undifferentiated	2558 (71.3%)	20830 (61.9%)	28538 (54.3%)	0.498	P<0.001
Grade	Unknown	648 (18.1%)	5477 (16.3%)	8200 (15.6%)		P<0.001
Grade	Well/moderately differentiated	382 (10.6%)	7336 (21.8%)	15829 (30.1%)		P<0.001
Summary stage	Distant	2090 (58.2%)	15350 (45.6%)	17145 (32.6%)	0.533	P<0.001
Summary stage	Localized	388 (10.8%)	5472 (16.3%)	13287 (25.3%)		P<0.001
Summary stage	Regional	967 (27.0%)	11305 (33.6%)	16224 (30.9%)		P<0.001
Summary stage	Unknown	143 (4.0%)	1516 (4.5%)	5911 (11.2%)		P<0.001
Surgery	No	2101 (58.6%)	17650 (52.5%)	29303 (55.7%)	0.127	P<0.001
Surgery	Unknown	46 (1.3%)	368 (1.1%)	480 (0.9%)		P<0.001
Surgery	Yes	1441 (40.2%)	15625 (46.4%)	22784 (43.3%)		P<0.001
Radiotherapy	No/Unknown	2703 (75.3%)	23440 (69.7%)	40720 (77.5%)	0.200	P<0.001
Radiotherapy	Refused	10 (0.3%)	262 (0.8%)	863 (1.6%)		P<0.001
Radiotherapy	Yes	875 (24.4%)	9941 (29.5%)	10984 (20.9%)		P<0.001
Chemotherapy	No/Unknown	937 (26.1%)	11798 (35.1%)	32484 (61.8%)	0.770	P<0.001
Chemotherapy	Yes	2651 (73.9%)	21845 (64.9%)	20083 (38.2%)		P<0.001

Values are n (%) unless otherwise stated. SMD is the maximum absolute pairwise standardized mean difference across age groups and is shown once per variable. P values derive from chi-square tests. Treatment variables are registry-coded; No/Unknown does not establish confirmed non-treatment.

AYA patients had a greater prevalence of signet-ring cell carcinoma (42.0%) and distant stage (58.2%) than middle-aged adults (25.6% and 45.6%) or older adults (14.9% and 32.6%). Chemotherapy was coded as Yes in 73.9%, 64.9%, and 38.2%, respectively. These registry-coded patterns are descriptive and do not establish treatment effects.

### 3.5 Survival outcomes and multivariable Cox models

The OS and conventional CSS cohorts included 89,622 and 88,109 patients, respectively. Reverse Kaplan-Meier median follow-up was 11.36 years (95% CI 11.19-11.55). Five-year OS was 20.9% for AYA, 24.0% for middle-aged adults, and 18.7% for older adults; corresponding conventional CSS was 22.5%, 26.9%, and 25.3% (**Figure 4**).

**Figure 4:**
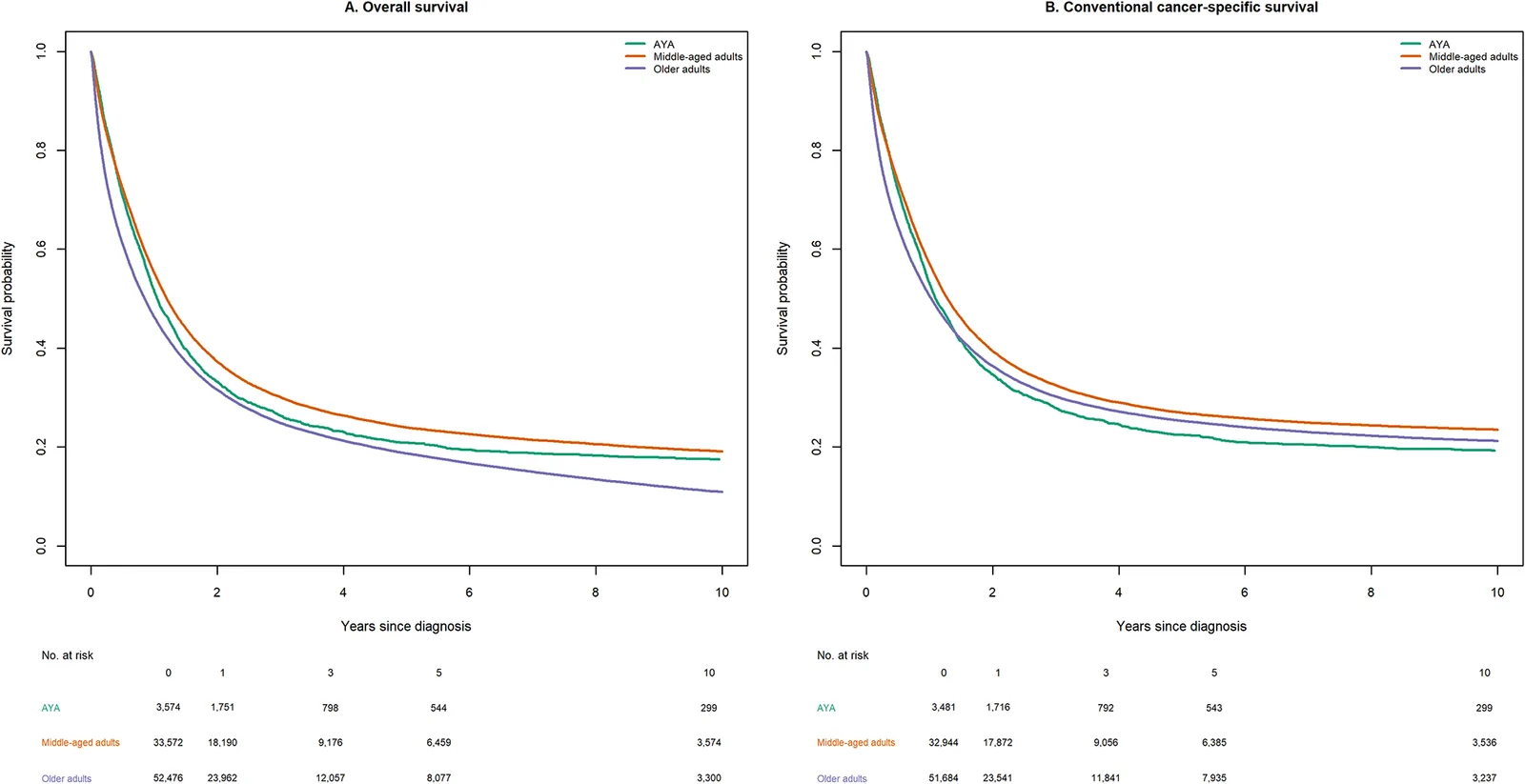
Ten-year SEER overall survival and conventional cancer-specific survival by age group. **(A, B)** show overall survival and conventional cancer-specific survival, respectively; numbers at risk are shown below each panel. Conventional cancer-specific survival censors non-cancer deaths.

Non-cancer deaths numbered 104 (2.9%), 2,494 (7.4%), and 9,206 (17.5%) among AYA, middle-aged, and older adults, respectively (**Supplementary Table S6**). CIFs distinguished gastric-cancer and non-cancer death (**Figure 5**); Gray tests were significant for both causes (P<0.001; **Supplementary Table S6**). In primary Model 2, compared with AYA, middle-aged and older adults had follow-up-averaged HRs of 1.11 (95% CI 1.06-1.15) and 1.73 (1.66-1.79) for all-cause death and 1.08 (1.04-1.13) and 1.52 (1.46-1.58) for gastric-cancer death, respectively (**Table 3**; **Figure 6**).

**Figure 5:**
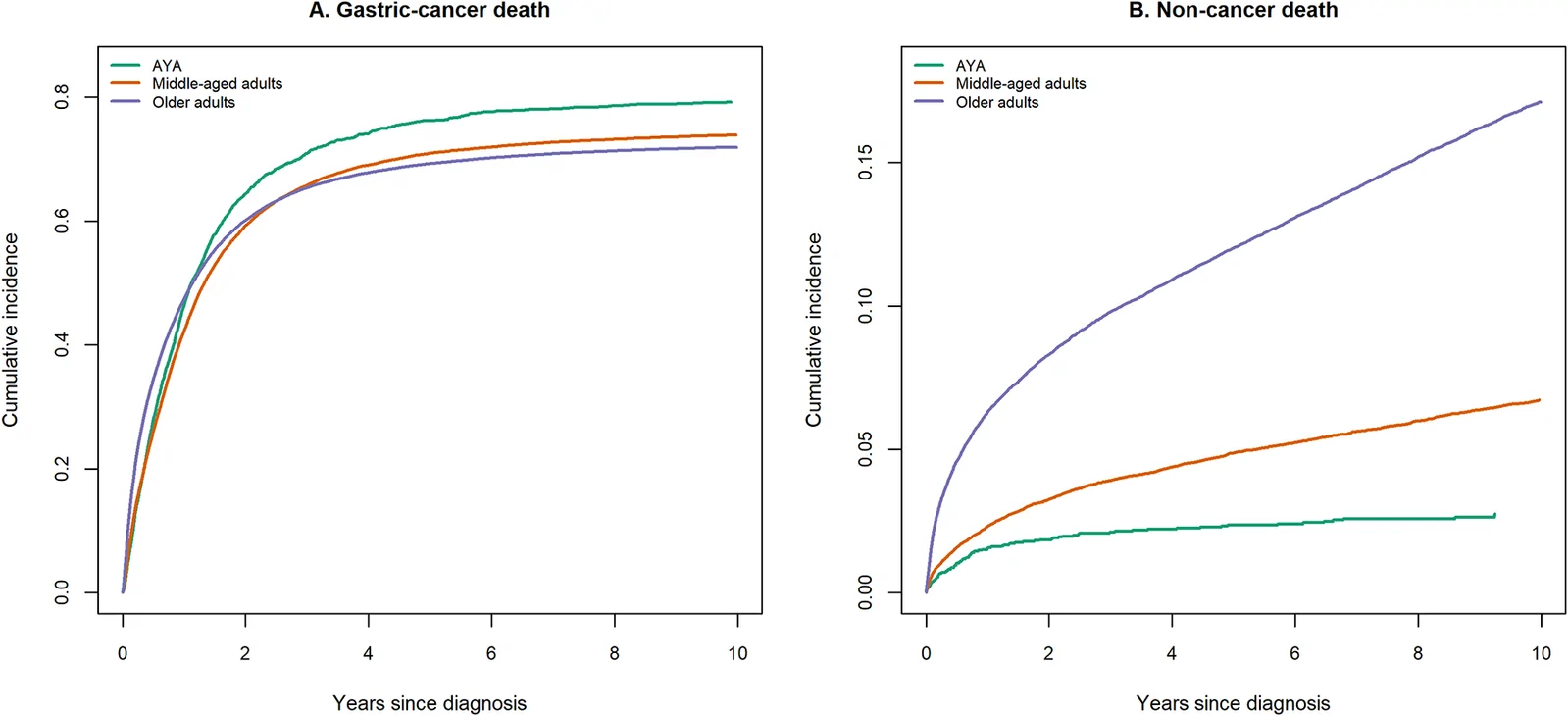
Cumulative incidence functions for gastric-cancer and non-cancer death by SEER age group. **(A)** Gastric-cancer death; **(B)** non-cancer death. Non-cancer death is treated as a competing event. Gray tests compare age groups.

**Table 3:** Primary clinicopathologic-adjusted Model 2 associations.

Comparison	OS HR (95% CI)	P value	Cause-specific HR (95% CI)	P value
Middle-aged adults vs AYA	1.11 (1.06-1.15)	P<0.001	1.08 (1.04-1.13)	P<0.001
Older adults vs AYA	1.73 (1.66-1.79)	P<0.001	1.52 (1.46-1.58)	P<0.001
Signet-ring cell carcinoma vs adenocarcinoma/NOS	1.06 (1.04-1.08)	P<0.001	1.09 (1.06-1.11)	P<0.001
Poorly/undifferentiated vs well/moderately differentiated	1.23 (1.21-1.25)	P<0.001	1.31 (1.29-1.34)	P<0.001
Regional vs localized stage	1.55 (1.51-1.58)	P<0.001	2.04 (1.98-2.10)	P<0.001
Distant vs localized stage	4.45 (4.35-4.55)	P<0.001	6.22 (6.05-6.39)	P<0.001

HR, hazard ratio; CI, confidence interval; AYA, adolescent and young adult. Fixed age HRs are follow-up-averaged associations because age violated proportional hazards. Model 2 adjusted for sex, race/ethnicity, diagnosis period, primary site, histologic subtype, grade, and stage.

**Figure 6:**
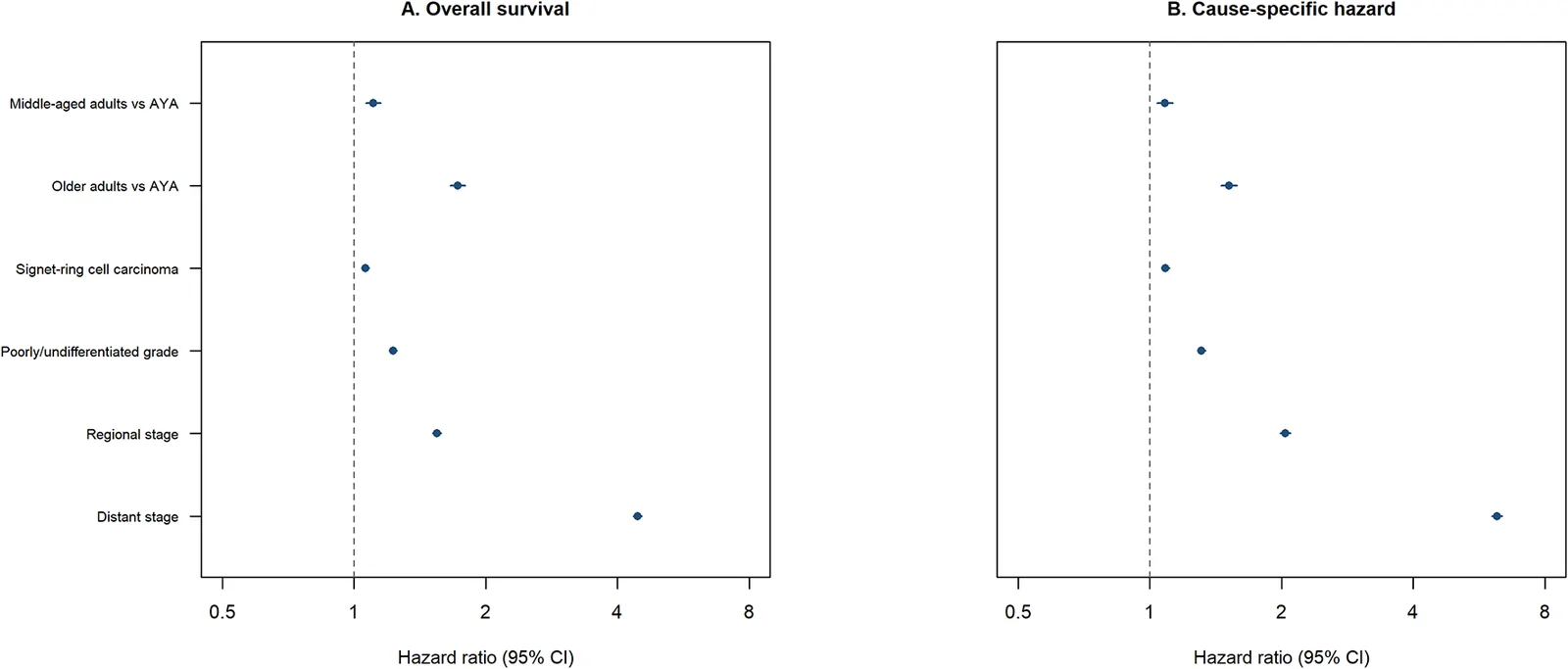
Primary clinicopathologic-adjusted Model 2 associations. **(A)** Overall survival; **(B)** cause-specific hazard for gastric-cancer death. AYA is the age reference. Points and lines show hazard ratios and 95% confidence intervals.

Full Model 1, primary Model 2, and exploratory Model 3 coefficients are provided in **Supplementary Tables S7-S9**. Formal PH diagnostics and secondary piecewise results are provided in **Supplementary Tables S10, S11** and **Supplementary Figures S3, S4**. Fixed age HRs are follow-up-averaged associations because age violated proportional hazards. Model 3 was exploratory and not interpreted as treatment effectiveness. Exact-zero exclusion sensitivity was robust (**Supplementary Table S12**). Detailed older-subgroup results are in **Supplementary Table S13** and **Supplementary Figure S5**.

## 4 Discussion

This complementary analysis of GBD 2021 and SEER evidence shows two related but distinct patterns in gastric cancer. At the population level, age-standardized incidence, death, and DALY rates declined during 1990-2021, but incident cases and deaths remained high and increased in absolute terms. At the patient level, SEER data showed that AYA, middle-aged, and older adults differed in clinicopathological characteristics, treatment patterns, OS, and CSS. Crude survival and competing-risk patterns differed across age groups. Older adults had poorer OS and substantially greater non-cancer mortality, whereas conventional CSS and gastric-cancer-death CIF patterns did not simply worsen monotonically with age. After clinicopathologic Model 2 adjustment, older age was associated with higher hazards of all-cause and gastric-cancer death, while AYA patients had higher proportions of signet-ring cell carcinoma and distant-stage disease. These findings are consistent with previous evidence showing that gastric cancer remains a major global health burden despite declining incidence or mortality rates in many settings (2, 3, 23).

The divergence between all-age numbers and age-standardized rates is important for interpretation. Incident cases increased from 980,899 to 1.23 million, and deaths increased from 854,185 to 954,374, while ASIR, ASDR, and the age-standardized DALY rate all declined. These estimates describe different aspects of burden. Numbers reflect the volume of patients and deaths that health systems must manage, whereas age-standardized rates provide a measure of standardized risk and outcome trends. Interpreting one without the other can understate either progress in standardized rates or the continuing service burden that must be managed (2, 3, 23).

Age provides the main interpretive link between population-level and patient-level findings. GBD identifies age-specific burden patterns, whereas SEER shows how age relates to clinical phenotype, competing mortality, and prognosis after diagnosis. These findings should not be interpreted as evidence that GBD age patterns cause SEER survival differences; the two sources provide complementary evidence from non-interchangeable populations.

AYA gastric cancer accounted for a small proportion of SEER cases, but the clinical profile of this group remains important. AYA patients had higher proportions of signet-ring cell carcinoma (42.0%) and distant-stage disease (58.2%) than the older groups (5, 6, 24). These patterns support careful clinical attention to persistent upper gastrointestinal symptoms in younger adults and argue against dismissing gastric cancer risk solely because of young age (24, 25). The data do not allow strong conclusions about hereditary or molecular mechanisms, because SEER lacks detailed molecular, germline, and family-history information (8, 25).

The SDI and GBD region results also show substantial heterogeneity. High-middle SDI had the highest 2021 ASIR, ASDR, and age-standardized DALY rate among SDI groups, while East Asia had the highest regional ASIR and age-standardized DALY rate and Andean Latin America had the highest regional ASDR. These differences may reflect variation in Helicobacter pylori prevalence, dietary exposures, smoking, screening practice, diagnostic access, treatment capacity, and the quality or completeness of input data used in GBD estimation (4, 23, 26). Because the GBD component was designed to contextualize global, SDI-level, and regional patterns rather than provide national benchmarking, country-level estimates, national rankings, country-level EAPC estimates, and maps were not included.

In the SEER models, treatment variables were added only in exploratory Model 3. Registry-coded ever-treatment may reflect timing, indication, stage, performance status, comorbidity, frailty, treatment intent, and access to care. Thus, Model 3 coefficients are not estimates of treatment benefit, effectiveness, or causal effects; chemotherapy No/Unknown cannot distinguish confirmed non-treatment from incomplete treatment information (27).

The findings support a two-level approach to gastric cancer control. At the population level, prevention, Helicobacter pylori management where appropriate, early diagnosis, and regional resource allocation remain important, particularly in settings with high age-standardized rates or large absolute burden (23, 28, 29). At the clinical level, age-aware management is needed. Younger adults may require earlier recognition when symptoms persist (24, 25), while older adults require comprehensive assessment that considers frailty, comorbidity, competing risks, treatment tolerance, and survivorship needs (10, 12, 30, 31). These implications should be viewed as direction-setting rather than prescriptive policy recommendations.

This study has several strengths and limitations. The complementary dual-source design juxtaposes global, SDI-level, and regional burden estimates from GBD with patient-level survival analyses from SEER, while keeping the two evidence streams analytically separate. Numbers and rates were treated as distinct measures, age-specific rates were not inappropriately aggregated, and sensitivity analyses were performed for key SEER modeling assumptions. Limitations include the modeled nature of GBD estimates, the absence of country-level data in the downloaded GBD dataset, the lack of individual linkage between GBD and SEER, and the limited global generalizability of SEER registry data (4, 14, 15). SEER also lacks comorbidity, frailty, performance status, molecular subtype, recurrence, and treatment-intent information. Treatment confounding, chemotherapy No/Unknown coding, stage and grade coding changes, and verified age non-proportional hazards further support interpreting the Cox estimates as follow-up-averaged Cox associations (22, 27).

## 5 Conclusion

This study showed that gastric cancer remains a substantial global and regional health burden despite declining age-standardized incidence, death, and DALY rates from 1990-2021. Complementary GBD 2021 and U.S. SEER evidence indicated that age is an important dimension of burden, clinical presentation, competing mortality, and post-diagnosis survival associations.

## Data Availability

Publicly available datasets were analyzed in this study. This data can be found here: GBD 2021 data are available through the Global Health Data Exchange and the GBD Results Tool: https://vizhub.healthdata.org/gbd-results/. SEER data are available through the Surveillance, Epidemiology, and End Results Program: https://seer.cancer.gov/data/ and were accessed using SEER*Stat after agreement to the SEER Research Data Use Agreement. No accession numbers are applicable.

## References

[1] Bray F Laversanne M Sung H Ferlay J Siegel RL Soerjomataram I . Global cancer statistics 2022: GLOBOCAN estimates of incidence and mortality worldwide for 36 cancers in 185 countries. CA Cancer J Clin. (2024) 74:229–63. doi: 10.3322/caac.21834 38572751

[2] Qin N Fan Y Yang T Yang Z Fan D . The burden of Gastric Cancer and possible risk factors from 1990 to 2021, and projections until 2035: findings from the Global Burden of Disease Study 2021. biomark Res. (2025) 13:5. doi: 10.1186/s40364-024-00720-8 39773334 PMC11708091

[3] Zhou L Han B Yuan Y Dong Z Shi Y Zheng R . The global burden of stomach cancer and its risk factors from 1990 to 2021: findings from the Global Burden of Disease Study 2021. BMC Public Health. (2025) 25:2678. doi: 10.1186/s12889-025-23901-y 40770796 PMC12326841

[4] GBD 2021 Diseases and Injuries Collaborators . Global incidence, prevalence, years lived with disability (YLDs), disability-adjusted life-years (DALYs), and healthy life expectancy (HALE) for 371 diseases and injuries in 204 countries and territories and 811 subnational locations, 1990-2021: a systematic analysis for the Global Burden of Disease Study 2021. Lancet. (2024) 403:2133–61. doi: 10.1016/S0140-6736(24)00757-8 PMC11122111 38642570

[5] Al-Refaie WB Hu CY Pisters PW Chang GJ . Gastric adenocarcinoma in young patients: a population-based appraisal. Ann Surg Oncol. (2011) 18:2800–7. doi: 10.1245/s10434-011-1647-x 21424881 PMC3266841

[6] Kist M Thomaschewski M Keck Y Abdalla TSA Zeissig SR Kleihues-van Tol K . Specifics of young gastric cancer patients: A population-based analysis of 46,110 patients with gastric cancer from the german clinical cancer registry group. Cancers (Basel). (2022) 14:5927. doi: 10.3390/cancers14235927 36497406 PMC9739447

[7] Panda SS Mohanty SS Sanyal A Ganesan P Kayal S Rathnam K . Adolescent and young adults with gastric cancer (AYA-GC)-the dilemma of an under-represented group: A multi-institutional analysis from the indian subcontinent. South Asian J Cancer. (2024) 13:142–5. doi: 10.1055/s-0043-1771275 PMC11196132 38919666

[8] Li J . Gastric cancer in young adults: A different clinical entity from carcinogenesis to prognosis. Gastroenterol Res Pract. (2020) 2020:9512707. doi: 10.1155/2020/9512707 32190044 PMC7071806

[9] Hydeman JA Uwazurike OC Adeyemi EI Beaupin LK . Survivorship needs of adolescent and young adult cancer survivors: a concept mapping analysis. J Cancer Surviv. (2019) 13:34–42. doi: 10.1007/s11764-018-0725-5 30604138 PMC6699632

[10] Joharatnam-Hogan N Shiu KK Khan K . Challenges in the treatment of gastric cancer in the older patient. Cancer Treat Rev. (2020) 85:101980. doi: 10.1016/j.ctrv.2020.101980 32065879

[11] Loizides S Papamichael D . Considerations and challenges in the management of the older patients with gastric cancer. Cancers (Basel). (2022) 14:1587. doi: 10.3390/cancers14061587 35326739 PMC8946244

[12] Tang WZ Tan ZK Qiu LY Chen JQ Jia K . Prevalence and unfavorable outcome of frailty in older adults with gastric cancer: a systematic review and meta-analysis. Support Care Cancer. (2024) 32:115. doi: 10.1007/s00520-024-08306-8 38240829

[13] Zhang T Zhang Y Leng X . Global, regional, and national trends in gastric cancer burden: 1990-2021 and projections to 2040. Front Oncol. (2024) 14:1468488. doi: 10.3389/fonc.2024.1468488 39726708 PMC11669584

[14] Murphy PK Sellers ME Bonds SH Scott S . The SEER Program's longstanding commitment to making cancer resources available. J Natl Cancer Inst Monogr. (2024) 2024:118–22. doi: 10.1093/jncimonographs/lgae028 39102882 PMC11300012

[15] Penberthy L Friedman S . The SEER Program's evolution: supporting clinically meaningful population-level research. J Natl Cancer Inst Monogr. (2024) 2024:110–7. doi: 10.1093/jncimonographs/lgae022 39102886 PMC11300003

[16] Tan CS Støer N Ning Y Chen Y Reilly M . Quantifying temporal trends of age-standardized rates with odds. Popul Health Metr. (2018) 16:18. doi: 10.1186/s12963-018-0173-5 30563536 PMC6299543

[17] Poythress JC Lee MY Young J . Planning and analyzing clinical trials with competing risks: Recommendations for choosing appropriate statistical methodology. Pharm Stat. (2020) 19:4–21. doi: 10.1002/pst.1966 31625290

[18] Austin PC . Balance diagnostics for comparing the distribution of baseline covariates between treatment groups in propensity-score matched samples. Stat Med. (2009) 28:3083–107. doi: 10.1002/sim.3697 19757444 PMC3472075

[19] Schemper M Smith TL . A note on quantifying follow-up in studies of failure time. Control Clin Trials. (1996) 17:343–6. doi: 10.1016/0197-2456(96)00075-x 8889347

[20] Clark TG Bradburn MJ Love SB Altman DG . Survival analysis part I: basic concepts and first analyses. Br J Cancer. (2003) 89:232–8. doi: 10.1038/sj.bjc.6601118 12865907 PMC2394262

[21] Bradburn MJ Clark TG Love SB Altman DG . Survival analysis part II: multivariate data analysis--an introduction to concepts and methods. Br J Cancer. (2003) 89:431–6. doi: 10.1038/sj.bjc.6601119 12888808 PMC2394368

[22] Bellera CA MacGrogan G Debled M de Lara CT Brouste V Mathoulin-Pélissier S . Variables with time-varying effects and the Cox model: some statistical concepts illustrated with a prognostic factor study in breast cancer. BMC Med Res Methodol. (2010) 10:20. doi: 10.1186/1471-2288-10-20 20233435 PMC2846954

[23] Inoue M . Epidemiology of gastric cancer-changing trends and global disparities. Cancers (Basel). (2024) 16:2948. doi: 10.3390/cancers16172948 39272806 PMC11394435

[24] Li Y Hahn AI Laszkowska M Jiang F Zauber AG Leung WK . Clinicopathological characteristics and risk factors of young-onset gastric carcinoma: A systematic review and meta-analysis. Clin Transl Gastroenterol. (2024) 15:e1. doi: 10.14309/ctg.0000000000000714 38717039 PMC11196083

[25] Michelon I Saeed A . Young-onset gastric cancer: clinical and genetic perspectives. J Gastric Cancer. (2026) 26:52–61. doi: 10.5230/jgc.2026.26.e9 41517847 PMC12802042

[26] Kim B Cho SJ . Endoscopic screening and surveillance for gastric cancer. Gastrointest Endosc Clin N Am. (2021) 31:489–501. doi: 10.1016/j.giec.2021.03.004 34053635

[27] Noone AM Lund JL Mariotto A Cronin K McNeel T Deapen D . Comparison of SEER treatment data with medicare claims. Med Care. (2016) 54:e55–64. doi: 10.1097/mlr.0000000000000073 24638121 PMC4981219

[28] Pan KF Li WQ Zhang L Liu WD Ma JL Zhang Y . Gastric cancer prevention by community eradication of Helicobacter pylori: a cluster-randomized controlled trial. Nat Med. (2024) 30:3250–60. doi: 10.1038/s41591-024-03153-w 39079993

[29] Ford AC Yuan Y Park JY Forman D Moayyedi P . Eradication therapy to prevent gastric cancer in helicobacterpylori-positive individuals: systematic review and meta-analysis of randomized controlled trials and observational studies. Gastroenterology. (2025) 169:261–76. doi: 10.1053/j.gastro.2024.12.033 39824392

[30] Paredero-Pérez I Jimenez-Fonseca P Cano JM Arrazubi V Carmona-Bayonas A Covela-Rúa M . State of the scientific evidence and recommendations for the management of older patients with gastric cancer. J Geriatr Oncol. (2024) 15:101657. doi: 10.1016/j.jgo.2023.101657 37957106

[31] Rodriguez-Arroyo B Caraballo P Pineda-Isaza A Arias-Valderrama O Cleves MA Zambrano AR . Geriatric assessment scale for optimal management of gastric cancer in older adults who underwent gastrectomy: A systematic review. Oncol Res Treat. (2024) 47:420–9. doi: 10.1159/000539774 38870920

