# Supplementary material for: Global and regional gastric cancer burden and age-related survival disparities: complementary analyses of GBD 2021 and SEER data
Source: Front Oncol. 2026 Oct 20;16:1880227. doi: 10.3389/fonc.2026.1880227 (PMC13727433; doi:10.3389/fonc.2026.1880227)
Supplement: Supplementary file 1 [file Supplementaryfile1.docx]

Supplementary Material

# 1 Supplementary Figures and Tables

## 1.1 Supplementary Figures

**Supplementary Figure 1.** SDI-specific GBD trends.

**Supplementary Figure 2.** GBD-region comparison.

**Supplementary Figure 3.** Formal proportional-hazards diagnostics for primary Model 2.

**Supplementary Figure 4.** Secondary interval-averaged piecewise age associations.

**Supplementary Figure 5.** Selected-time survival and competing-risk estimates among older subgroups.

## 1.2 Supplementary Tables

**Supplementary Table 1.** SEER cohort eligibility and ICD-O-3 morphology mapping.

| Criterion | Definition |
| --- | --- |
| Primary site | Stomach C16.x |
| Behavior | Malignant |
| Diagnosis period | 2000–2021 |
| Age | ≥15 years, known age |
| Sex | Male or female |
| Primary sequence | One primary tumor only or first of multiple primary tumors |
| Prespecified morphology source range | 8140–8239 or 8250–8579 |
| Morphology refinement | SEER Rare Cancer Classification 8.1 |
| Final adenocarcinoma-focused analytic cohort | N = 89,798 |

Note: This table presents the prespecified cohort eligibility criteria and the observed morphology codes used after adenocarcinoma morphology refinement.

**Supplementary Table 2.** SDI-specific GBD burden and temporal trends.

| SDI group | Outcome | 1990 number (95% UI) | 2021 number (95% UI) | 1990 age-standardized rate (95% UI) | 2021 age-standardized rate (95% UI) | EAPC (95% CI) |
| --- | --- | --- | --- | --- | --- | --- |
| High SDI | Incidence | 254,259 (242,222–261,672) | 239,119 (215,071–256,938) | 23.13 (22.05–23.80) | 11.16 (10.21–11.91) | -2.40% (-2.46% to -2.34%) |
| High SDI | Deaths | 175,683 (166,255–181,197) | 153,539 (136,670–165,354) | 15.86 (15.01–16.37) | 6.83 (6.18–7.34) | -2.79% (-2.81% to -2.76%) |
| High SDI | DALYs | 4,099,422 (3,902,053–4,218,897) | 2,897,404 (2,654,584–3,101,177) | 381.13 (362.21–392.23) | 146.10 (135.56–155.89) | -3.15% (-3.18% to -3.13%) |
| High-middle SDI | Incidence | 332,614 (298,475–360,843) | 387,196 (315,957–457,286) | 33.33 (30.09–36.10) | 19.62 (16.02–23.13) | -1.75% (-1.84% to -1.65%) |
| High-middle SDI | Deaths | 304,773 (274,911–330,960) | 295,105 (244,787–343,611) | 31.08 (28.09–33.70) | 14.93 (12.40–17.36) | -2.47% (-2.59% to -2.35%) |
| High-middle SDI | DALYs | 8,241,238 (7,281,203–9,010,822) | 6,901,607 (5,703,610–8,141,888) | 802.75 (711.79–876.76) | 353.18 (291.89–416.78) | -2.79% (-2.91% to -2.66%) |
| Middle SDI | Incidence | 303,889 (262,410–351,115) | 451,465 (368,224–542,292) | 28.89 (25.13–33.44) | 16.91 (13.79–20.28) | -1.79% (-1.88% to -1.70%) |
| Middle SDI | Deaths | 284,016 (246,760–329,474) | 356,459 (294,160–423,770) | 28.10 (24.61–32.65) | 13.72 (11.31–16.22) | -2.42% (-2.54% to -2.30%) |
| Middle SDI | DALYs | 8,208,596 (7,034,443–9,506,074) | 8,820,717 (7,336,337–10,567,624) | 721.81 (624.06–835.38) | 320.24 (266.07–382.87) | -2.75% (-2.86% to -2.63%) |
| Low-middle SDI | Incidence | 63,086 (55,336–76,805) | 110,396 (97,098–126,222) | 10.14 (8.98–12.42) | 7.68 (6.71–8.77) | -0.82% (-0.86% to -0.78%) |
| Low-middle SDI | Deaths | 62,598 (54,993–76,278) | 107,456 (94,064–122,211) | 10.45 (9.25–12.79) | 7.71 (6.69–8.76) | -0.89% (-0.94% to -0.85%) |
| Low-middle SDI | DALYs | 1,874,805 (1,629,366–2,261,562) | 2,953,125 (2,599,859–3,357,038) | 274.45 (239.89–333.49) | 192.56 (169.42–219.14) | -1.08% (-1.12% to -1.04%) |
| Low SDI | Incidence | 26,334 (20,975–30,114) | 41,388 (32,600–46,956) | 11.41 (9.04–13.03) | 8.13 (6.44–9.22) | -1.11% (-1.16% to -1.06%) |
| Low SDI | Deaths | 26,410 (21,016–30,213) | 41,192 (32,515–46,864) | 11.90 (9.41–13.57) | 8.46 (6.71–9.60) | -1.10% (-1.15% to -1.04%) |
| Low SDI | DALYs | 795,640 (636,078–910,967) | 1,198,903 (940,407–1,371,290) | 311.98 (248.62–357.31) | 209.77 (165.60–238.95) | -1.35% (-1.40% to -1.29%) |

Note: GBD 2021 estimates. 95% UI denotes the GBD uncertainty interval; 95% CI denotes the regression confidence interval for EAPC. Rates are per 100,000 population.

**Supplementary Table 3.** GBD region-specific burden and temporal trends.

| GBD region | Outcome | 1990 number (95% UI) | 2021 number (95% UI) | 1990 age-standardized rate (95% UI) | 2021 age-standardized rate (95% UI) | EAPC (95% CI) |
| --- | --- | --- | --- | --- | --- | --- |
| Oceania | Incidence | 513 (364–662) | 1,009 (787–1,258) | 17.12 (12.57–21.85) | 13.26 (10.49–16.34) | -0.84% (-0.91% to -0.78%) |
| Oceania | Deaths | 497 (358–641) | 957 (744–1,201) | 17.76 (13.08–22.53) | 13.45 (10.67–16.61) | -0.90% (-0.97% to -0.83%) |
| Oceania | DALYs | 15,969 (11,130–20,718) | 30,296 (23,279–38,275) | 456.41 (329.10–588.19) | 341.81 (265.80–428.05) | -0.93% (-1.02% to -0.85%) |
| Tropical Latin America | Incidence | 16,863 (16,115–17,487) | 25,611 (23,899–26,903) | 18.91 (17.91–19.68) | 9.99 (9.30–10.51) | -2.16% (-2.22% to -2.11%) |
| Tropical Latin America | Deaths | 16,803 (16,029–17,446) | 24,867 (23,070–26,204) | 19.54 (18.45–20.37) | 9.78 (9.05–10.32) | -2.31% (-2.37% to -2.26%) |
| Tropical Latin America | DALYs | 449,287 (432,709–464,461) | 619,369 (586,946–648,930) | 463.86 (444.88–480.64) | 237.81 (225.12–249.26) | -2.28% (-2.33% to -2.22%) |
| Southeast Asia | Incidence | 28,024 (22,597–32,063) | 47,761 (41,101–56,374) | 10.72 (8.69–12.28) | 7.27 (6.28–8.69) | -1.45% (-1.52% to -1.38%) |
| Southeast Asia | Deaths | 27,448 (22,187–31,331) | 43,303 (37,510–51,160) | 10.92 (8.91–12.54) | 6.83 (5.94–8.16) | -1.70% (-1.77% to -1.63%) |
| Southeast Asia | DALYs | 820,413 (659,024–941,613) | 1,193,413 (1,021,566–1,404,696) | 285.40 (229.89–326.55) | 170.78 (146.62–201.09) | -1.83% (-1.90% to -1.76%) |
| South Asia | Incidence | 46,713 (39,946–59,247) | 84,803 (73,745–102,263) | 7.71 (6.59–9.93) | 5.70 (4.92–6.84) | -0.90% (-0.99% to -0.82%) |
| South Asia | Deaths | 46,010 (39,093–58,610) | 82,924 (71,235–99,367) | 7.88 (6.68–10.17) | 5.72 (4.91–6.84) | -0.94% (-1.03% to -0.86%) |
| South Asia | DALYs | 1,432,660 (1,232,646–1,789,557) | 2,295,875 (2,004,284–2,773,948) | 215.57 (183.72–273.18) | 145.55 (126.31–175.43) | -1.21% (-1.28% to -1.14%) |
| East Asia | Incidence | 415,521 (345,214–486,249) | 624,688 (483,569–778,629) | 47.14 (39.54–55.52) | 28.64 (22.23–35.55) | -1.63% (-1.80% to -1.47%) |
| East Asia | Deaths | 381,435 (318,259–449,024) | 455,947 (355,895–567,156) | 45.18 (38.27–53.36) | 21.26 (16.59–26.22) | -2.52% (-2.73% to -2.31%) |
| East Asia | DALYs | 10,985,613 (9,060,114–12,871,606) | 10,921,589 (8,491,323–13,670,612) | 1160.15 (965.28–1363.00) | 496.94 (386.19–619.66) | -2.87% (-3.05% to -2.68%) |
| Central Latin America | Incidence | 16,300 (15,735–16,774) | 29,824 (26,593–33,520) | 20.19 (19.33–20.81) | 11.98 (10.69–13.44) | -1.96% (-2.04% to -1.88%) |
| Central Latin America | Deaths | 16,459 (15,832–16,952) | 28,763 (25,594–32,289) | 21.14 (20.17–21.80) | 11.69 (10.41–13.09) | -2.17% (-2.25% to -2.09%) |
| Central Latin America | DALYs | 427,467 (414,311–439,042) | 722,803 (644,910–813,721) | 482.23 (466.65–496.63) | 282.56 (252.21–317.88) | -1.99% (-2.07% to -1.90%) |
| Central Sub-Saharan Africa | Incidence | 2,568 (1,894–3,121) | 4,664 (3,482–5,777) | 11.51 (8.58–13.89) | 8.54 (6.39–10.51) | -1.01% (-1.05% to -0.97%) |
| Central Sub-Saharan Africa | Deaths | 2,570 (1,888–3,136) | 4,607 (3,474–5,734) | 12.13 (9.06–14.67) | 8.93 (6.76–11.06) | -1.03% (-1.07% to -0.99%) |
| Central Sub-Saharan Africa | DALYs | 77,401 (57,088–94,838) | 139,267 (104,199–175,157) | 305.95 (225.60–372.66) | 220.04 (165.52–273.38) | -1.11% (-1.14% to -1.07%) |
| Caribbean | Incidence | 3,132 (2,870–3,439) | 4,279 (3,708–4,900) | 12.15 (11.12–13.32) | 7.97 (6.91–9.13) | -1.28% (-1.35% to -1.21%) |
| Caribbean | Deaths | 3,138 (2,873–3,450) | 4,191 (3,626–4,808) | 12.38 (11.34–13.57) | 7.79 (6.74–8.94) | -1.41% (-1.48% to -1.33%) |
| Caribbean | DALYs | 79,115 (71,374–87,961) | 104,402 (89,158–120,825) | 296.37 (268.46–329.40) | 195.67 (167.08–226.69) | -1.25% (-1.35% to -1.16%) |
| North Africa and Middle East | Incidence | 24,709 (18,612–27,925) | 42,569 (29,749–48,466) | 14.71 (11.07–16.60) | 9.67 (6.82–10.95) | -1.25% (-1.34% to -1.16%) |
| North Africa and Middle East | Deaths | 24,484 (18,566–27,726) | 39,717 (27,883–45,110) | 15.19 (11.51–17.15) | 9.43 (6.70–10.67) | -1.42% (-1.51% to -1.33%) |
| North Africa and Middle East | DALYs | 707,934 (531,826–799,708) | 1,057,332 (732,444–1,215,400) | 380.20 (288.74–429.88) | 217.41 (151.81–247.77) | -1.75% (-1.82% to -1.67%) |
| Andean Latin America | Incidence | 6,287 (5,533–7,155) | 12,593 (10,226–15,421) | 31.13 (27.43–35.50) | 21.48 (17.41–26.27) | -1.41% (-1.56% to -1.26%) |
| Andean Latin America | Deaths | 6,455 (5,696–7,324) | 12,368 (10,039–15,114) | 32.80 (28.93–37.32) | 21.33 (17.30–26.08) | -1.59% (-1.74% to -1.44%) |
| Andean Latin America | DALYs | 168,541 (147,325–190,607) | 292,675 (235,472–359,119) | 773.16 (678.85–875.03) | 485.41 (391.23–595.07) | -1.73% (-1.88% to -1.59%) |
| High-income Asia Pacific | Incidence | 130,992 (123,589–135,804) | 123,451 (107,133–133,459) | 65.16 (61.41–67.66) | 25.43 (22.80–27.27) | -3.08% (-3.15% to -3.01%) |
| High-income Asia Pacific | Deaths | 72,724 (67,979–75,561) | 70,689 (60,268–77,102) | 37.06 (34.46–38.60) | 13.13 (11.52–14.14) | -3.42% (-3.46% to -3.37%) |
| High-income Asia Pacific | DALYs | 1,831,592 (1,693,439–1,906,727) | 1,194,021 (1,061,152–1,287,243) | 900.48 (832.19–937.87) | 273.07 (250.22–294.45) | -3.91% (-3.95% to -3.86%) |
| Australasia | Incidence | 2,409 (2,259–2,538) | 3,187 (2,823–3,478) | 10.26 (9.63–10.83) | 5.93 (5.32–6.43) | -1.80% (-1.89% to -1.72%) |
| Australasia | Deaths | 1,804 (1,690–1,906) | 2,191 (1,921–2,388) | 7.73 (7.25–8.16) | 3.91 (3.49–4.25) | -2.19% (-2.32% to -2.06%) |
| Australasia | DALYs | 39,867 (37,637–41,910) | 42,612 (38,796–46,055) | 171.48 (161.92–180.59) | 84.88 (77.89–91.35) | -2.26% (-2.38% to -2.15%) |
| Central Asia | Incidence | 12,594 (11,989–13,286) | 9,763 (8,797–10,942) | 26.36 (25.05–27.82) | 11.78 (10.66–13.11) | -2.34% (-2.45% to -2.24%) |
| Central Asia | Deaths | 12,366 (11,762–13,059) | 9,332 (8,400–10,425) | 26.28 (24.93–27.77) | 11.56 (10.46–12.83) | -2.39% (-2.50% to -2.29%) |
| Central Asia | DALYs | 359,741 (343,480–378,737) | 264,445 (236,310–298,863) | 720.77 (687.22–760.05) | 298.62 (268.02–336.43) | -2.66% (-2.74% to -2.59%) |
| Central Europe | Incidence | 27,272 (26,183–28,244) | 20,269 (18,700–21,903) | 18.34 (17.57–19.01) | 9.19 (8.49–9.94) | -2.30% (-2.40% to -2.21%) |
| Central Europe | Deaths | 27,224 (26,133–28,216) | 19,182 (17,694–20,668) | 18.50 (17.72–19.19) | 8.54 (7.89–9.22) | -2.60% (-2.68% to -2.51%) |
| Central Europe | DALYs | 666,468 (641,593–689,338) | 418,848 (387,111–452,190) | 444.41 (427.88–459.51) | 201.30 (185.93–217.26) | -2.64% (-2.73% to -2.55%) |
| Southern Latin America | Incidence | 8,563 (8,077–9,055) | 9,965 (9,007–10,841) | 18.70 (17.61–19.79) | 11.39 (10.32–12.38) | -1.43% (-1.55% to -1.32%) |
| Southern Latin America | Deaths | 8,435 (7,955–8,911) | 9,292 (8,379–10,092) | 18.65 (17.56–19.75) | 10.51 (9.47–11.41) | -1.67% (-1.78% to -1.56%) |
| Southern Latin America | DALYs | 201,215 (190,126–212,735) | 205,763 (188,547–223,240) | 432.39 (408.29–457.03) | 241.14 (221.13–261.57) | -1.71% (-1.82% to -1.60%) |
| High-income North America | Incidence | 29,244 (27,507–30,219) | 33,113 (30,756–34,704) | 8.30 (7.84–8.56) | 5.23 (4.90–5.46) | -1.56% (-1.60% to -1.51%) |
| High-income North America | Deaths | 19,836 (18,496–20,571) | 19,363 (17,658–20,393) | 5.57 (5.21–5.77) | 2.95 (2.72–3.10) | -2.12% (-2.17% to -2.06%) |
| High-income North America | DALYs | 438,698 (419,948–450,664) | 419,798 (396,159–437,428) | 129.31 (124.20–132.72) | 70.88 (67.42–73.54) | -1.97% (-2.04% to -1.90%) |
| Western Europe | Incidence | 99,469 (93,628–102,944) | 73,911 (65,944–78,835) | 16.99 (16.04–17.56) | 7.81 (7.11–8.25) | -2.45% (-2.50% to -2.40%) |
| Western Europe | Deaths | 86,059 (80,686–89,117) | 56,322 (49,569–60,192) | 14.52 (13.64–15.03) | 5.57 (5.02–5.90) | -3.08% (-3.17% to -2.99%) |
| Western Europe | DALYs | 1,769,700 (1,688,901–1,823,382) | 1,038,057 (950,524–1,095,124) | 316.11 (302.86–324.92) | 120.38 (112.31–126.18) | -3.08% (-3.16% to -3.00%) |
| Eastern Europe | Incidence | 92,743 (90,216–94,582) | 51,006 (46,933–54,984) | 32.94 (31.98–33.58) | 14.67 (13.48–15.85) | -2.81% (-2.90% to -2.72%) |
| Eastern Europe | Deaths | 83,348 (81,061–85,100) | 42,700 (39,316–46,074) | 29.71 (28.85–30.32) | 12.15 (11.17–13.12) | -3.11% (-3.21% to -3.02%) |
| Eastern Europe | DALYs | 2,264,040 (2,215,067–2,307,995) | 1,031,465 (943,209–1,119,000) | 805.45 (787.50–821.16) | 306.55 (280.01–332.59) | -3.40% (-3.52% to -3.29%) |
| Southern Sub-Saharan Africa | Incidence | 2,178 (1,715–2,438) | 4,055 (3,376–4,570) | 7.92 (6.21–8.87) | 7.02 (5.80–7.86) | -0.43% (-0.75% to -0.10%) |
| Southern Sub-Saharan Africa | Deaths | 2,151 (1,697–2,411) | 3,967 (3,307–4,472) | 8.13 (6.38–9.11) | 7.13 (5.92–7.98) | -0.47% (-0.81% to -0.13%) |
| Southern Sub-Saharan Africa | DALYs | 62,836 (49,869–70,324) | 111,785 (94,036–128,129) | 208.13 (164.69–233.47) | 177.66 (148.51–201.91) | -0.50% (-0.84% to -0.15%) |
| Western Sub-Saharan Africa | Incidence | 6,579 (5,585–7,728) | 12,070 (9,336–14,239) | 7.58 (6.45–8.96) | 6.33 (5.01–7.47) | -0.37% (-0.45% to -0.30%) |
| Western Sub-Saharan Africa | Deaths | 6,705 (5,699–7,866) | 12,151 (9,468–14,304) | 8.01 (6.85–9.50) | 6.69 (5.34–7.90) | -0.36% (-0.44% to -0.29%) |
| Western Sub-Saharan Africa | DALYs | 186,729 (157,190–218,063) | 338,256 (257,059–405,471) | 196.52 (166.83–230.65) | 156.08 (120.91–184.46) | -0.55% (-0.62% to -0.48%) |
| Eastern Sub-Saharan Africa | Incidence | 8,228 (6,526–9,433) | 11,642 (9,555–13,652) | 10.65 (8.47–12.15) | 6.80 (5.65–7.95) | -1.68% (-1.76% to -1.60%) |
| Eastern Sub-Saharan Africa | Deaths | 8,233 (6,531–9,437) | 11,542 (9,518–13,490) | 11.09 (8.81–12.70) | 7.09 (5.92–8.28) | -1.66% (-1.74% to -1.58%) |
| Eastern Sub-Saharan Africa | DALYs | 252,008 (199,160–287,489) | 344,564 (281,270–404,252) | 292.75 (232.08–335.22) | 175.48 (144.38–205.24) | -1.90% (-1.99% to -1.82%) |

Note: GBD 2021 estimates. 95% UI denotes the GBD uncertainty interval; 95% CI denotes the regression confidence interval for EAPC. Rates are per 100,000 population.

**Supplementary Table 4.** Age- and sex-specific GBD burden in 1990 and 2021.

| Sex | Age group | Metric | 1990 estimate (95% UI) | 2021 estimate (95% UI) |
| --- | --- | --- | --- | --- |
| Male | 15-19 years | Number | 764 (595–899) | 380 (279–448) |
| Female | 15-19 years | Number | 640 (560–711) | 347 (309–385) |
| Both | 15-19 years | Number | 1,405 (1,183–1,551) | 728 (601–810) |
| Male | 15-19 years | Rate | 0.29 (0.23–0.34) | 0.12 (0.09–0.14) |
| Female | 15-19 years | Rate | 0.25 (0.22–0.28) | 0.11 (0.10–0.13) |
| Both | 15-19 years | Rate | 0.27 (0.23–0.30) | 0.12 (0.10–0.13) |
| Male | 20-24 years | Number | 1,654 (1,266–1,916) | 1,033 (783–1,186) |
| Female | 20-24 years | Number | 1,649 (1,429–1,911) | 1,004 (876–1,119) |
| Both | 20-24 years | Number | 3,303 (2,867–3,631) | 2,038 (1,745–2,240) |
| Male | 20-24 years | Rate | 0.67 (0.51–0.77) | 0.34 (0.26–0.39) |
| Female | 20-24 years | Rate | 0.68 (0.59–0.78) | 0.34 (0.30–0.38) |
| Both | 20-24 years | Rate | 0.67 (0.58–0.74) | 0.34 (0.29–0.38) |
| Male | 25-29 years | Number | 3,359 (2,715–3,777) | 2,804 (2,150–3,226) |
| Female | 25-29 years | Number | 3,624 (3,187–4,126) | 2,477 (2,162–2,779) |
| Both | 25-29 years | Number | 6,983 (6,147–7,634) | 5,281 (4,464–5,798) |
| Male | 25-29 years | Rate | 1.51 (1.22–1.70) | 0.94 (0.72–1.08) |
| Female | 25-29 years | Rate | 1.65 (1.45–1.87) | 0.85 (0.74–0.95) |
| Both | 25-29 years | Rate | 1.58 (1.39–1.72) | 0.90 (0.76–0.99) |
| Male | 30-34 years | Number | 7,848 (6,318–8,885) | 7,969 (6,180–9,388) |
| Female | 30-34 years | Number | 6,400 (5,685–7,201) | 5,249 (4,600–6,024) |
| Both | 30-34 years | Number | 14,248 (12,464–15,517) | 13,217 (11,232–14,851) |
| Male | 30-34 years | Rate | 4.02 (3.23–4.55) | 2.61 (2.02–3.07) |
| Female | 30-34 years | Rate | 3.37 (2.99–3.79) | 1.76 (1.54–2.02) |
| Both | 30-34 years | Rate | 3.70 (3.23–4.03) | 2.19 (1.86–2.46) |
| Male | 35-39 years | Number | 16,602 (12,898–18,976) | 12,938 (10,134–15,572) |
| Female | 35-39 years | Number | 11,098 (9,778–12,569) | 7,837 (6,929–8,870) |
| Both | 35-39 years | Number | 27,700 (23,852–30,318) | 20,776 (17,794–23,551) |
| Male | 35-39 years | Rate | 9.29 (7.21–10.61) | 4.57 (3.58–5.50) |
| Female | 35-39 years | Rate | 6.40 (5.64–7.25) | 2.82 (2.49–3.19) |
| Both | 35-39 years | Rate | 7.86 (6.77–8.61) | 3.70 (3.17–4.20) |
| Male | 40-44 years | Number | 26,605 (21,404–30,204) | 21,409 (17,468–26,141) |
| Female | 40-44 years | Number | 14,160 (12,657–15,712) | 10,935 (9,756–12,236) |
| Both | 40-44 years | Number | 40,766 (35,070–44,607) | 32,344 (27,977–37,370) |
| Male | 40-44 years | Rate | 18.19 (14.63–20.65) | 8.49 (6.93–10.37) |
| Female | 40-44 years | Rate | 10.10 (9.03–11.21) | 4.41 (3.93–4.93) |
| Both | 40-44 years | Rate | 14.23 (12.24–15.57) | 6.47 (5.59–7.47) |
| Male | 45-49 years | Number | 35,833 (28,848–41,211) | 35,251 (28,896–44,376) |
| Female | 45-49 years | Number | 16,131 (14,528–17,799) | 15,487 (13,721–17,590) |
| Both | 45-49 years | Number | 51,964 (44,790–57,381) | 50,737 (43,456–60,094) |
| Male | 45-49 years | Rate | 30.27 (24.37–34.81) | 14.82 (12.15–18.66) |
| Female | 45-49 years | Rate | 14.17 (12.77–15.64) | 6.57 (5.82–7.46) |
| Both | 45-49 years | Rate | 22.38 (19.29–24.71) | 10.72 (9.18–12.69) |
| Male | 50-54 years | Number | 61,370 (49,771–70,208) | 61,916 (49,830–78,617) |
| Female | 50-54 years | Number | 24,899 (22,246–27,402) | 23,566 (20,629–27,179) |
| Both | 50-54 years | Number | 86,269 (74,413–95,433) | 85,483 (72,588–102,838) |
| Male | 50-54 years | Rate | 57.01 (46.23–65.22) | 27.89 (22.45–35.42) |
| Female | 50-54 years | Rate | 23.73 (21.20–26.12) | 10.57 (9.25–12.19) |
| Both | 50-54 years | Rate | 40.58 (35.01–44.89) | 19.21 (16.31–23.11) |
| Male | 55-59 years | Number | 84,426 (70,640–97,052) | 90,071 (73,472–111,780) |
| Female | 55-59 years | Number | 33,285 (29,759–37,433) | 33,162 (28,759–38,480) |
| Both | 55-59 years | Number | 117,711 (102,785–129,561) | 123,232 (104,531–145,418) |
| Male | 55-59 years | Rate | 90.89 (76.05–104.48) | 46.26 (37.73–57.40) |
| Female | 55-59 years | Rate | 36.06 (32.24–40.55) | 16.50 (14.31–19.14) |
| Both | 55-59 years | Rate | 63.56 (55.50–69.96) | 31.14 (26.41–36.75) |
| Male | 60-64 years | Number | 99,195 (85,563–111,477) | 101,429 (83,045–123,562) |
| Female | 60-64 years | Number | 42,826 (38,556–47,100) | 38,311 (34,298–43,358) |
| Both | 60-64 years | Number | 142,021 (127,514–154,893) | 139,740 (120,440–161,958) |
| Male | 60-64 years | Rate | 126.29 (108.93–141.92) | 65.21 (53.39–79.44) |
| Female | 60-64 years | Rate | 52.19 (46.98–57.40) | 23.29 (20.85–26.36) |
| Both | 60-64 years | Rate | 88.43 (79.39–96.44) | 43.66 (37.63–50.60) |
| Male | 65-69 years | Number | 95,440 (82,977–108,497) | 132,333 (108,181–159,481) |
| Female | 65-69 years | Number | 48,900 (44,688–53,574) | 52,820 (46,260–60,297) |
| Both | 65-69 years | Number | 144,340 (131,866–158,602) | 185,153 (157,412–214,943) |
| Male | 65-69 years | Rate | 166.47 (144.73–189.25) | 100.38 (82.06–120.97) |
| Female | 65-69 years | Rate | 73.78 (67.42–80.83) | 36.68 (32.12–41.87) |
| Both | 65-69 years | Rate | 116.77 (106.68–128.31) | 67.12 (57.07–77.92) |
| Male | 70-74 years | Number | 80,995 (71,291–93,611) | 134,817 (107,749–162,037) |
| Female | 70-74 years | Number | 47,157 (42,706–51,869) | 57,198 (49,836–65,549) |
| Both | 70-74 years | Number | 128,152 (117,220–142,419) | 192,015 (162,607–221,305) |
| Male | 70-74 years | Rate | 215.31 (189.51–248.84) | 139.86 (111.78–168.10) |
| Female | 70-74 years | Rate | 100.24 (90.78–110.26) | 52.26 (45.53–59.89) |
| Both | 70-74 years | Rate | 151.37 (138.46–168.22) | 93.28 (79.00–107.51) |
| Male | 75-79 years | Number | 62,124 (56,217–71,057) | 99,478 (80,499–118,584) |
| Female | 75-79 years | Number | 46,822 (42,658–50,301) | 49,755 (41,913–56,498) |
| Both | 75-79 years | Number | 108,946 (101,239–119,319) | 149,233 (126,471–169,400) |
| Male | 75-79 years | Rate | 246.20 (222.79–281.60) | 166.39 (134.64–198.34) |
| Female | 75-79 years | Rate | 128.90 (117.44–138.48) | 69.01 (58.13–78.36) |
| Both | 75-79 years | Rate | 176.99 (164.47–193.84) | 113.15 (95.90–128.45) |
| Male | 80-84 years | Number | 34,211 (30,501–39,475) | 70,844 (57,317–81,201) |
| Female | 80-84 years | Number | 32,130 (27,830–34,797) | 44,376 (35,794–50,755) |
| Both | 80-84 years | Number | 66,341 (59,900–72,235) | 115,220 (97,519–128,648) |
| Male | 80-84 years | Rate | 257.54 (229.62–297.18) | 193.29 (156.38–221.55) |
| Female | 80-84 years | Rate | 145.43 (125.97–157.51) | 87.13 (70.28–99.65) |
| Both | 80-84 years | Rate | 187.53 (169.32–204.19) | 131.56 (111.35–146.89) |
| Male | 85-89 years | Number | 13,931 (12,449–15,523) | 43,054 (35,311–48,605) |
| Female | 85-89 years | Number | 16,620 (13,784–18,174) | 31,986 (24,431–36,918) |
| Both | 85-89 years | Number | 30,551 (26,580–33,046) | 75,040 (61,735–83,880) |
| Male | 85-89 years | Rate | 275.09 (245.83–306.53) | 249.55 (204.67–281.73) |
| Female | 85-89 years | Rate | 165.42 (137.19–180.89) | 112.35 (85.82–129.68) |
| Both | 85-89 years | Rate | 202.18 (175.89–218.69) | 164.12 (135.02–183.46) |
| Male | 90-94 years | Number | 3,316 (2,919–3,682) | 14,426 (12,004–16,272) |
| Female | 90-94 years | Number | 5,293 (4,240–5,871) | 16,866 (12,145–19,599) |
| Both | 90-94 years | Number | 8,608 (7,204–9,399) | 31,292 (25,089–35,222) |
| Male | 90-94 years | Rate | 263.37 (231.89–292.44) | 247.51 (205.96–279.17) |
| Female | 90-94 years | Rate | 174.89 (140.12–194.00) | 139.84 (100.70–162.50) |
| Both | 90-94 years | Rate | 200.88 (168.11–219.33) | 174.92 (140.25–196.89) |
| Male | 95+ years | Number | 486 (395–536) | 2,769 (2,094–3,133) |
| Female | 95+ years | Number | 1,107 (828–1,260) | 5,937 (4,008–6,989) |
| Both | 95+ years | Number | 1,593 (1,219–1,783) | 8,705 (6,135–10,063) |
| Male | 95+ years | Rate | 186.88 (151.73–205.85) | 183.12 (138.46–207.21) |
| Female | 95+ years | Rate | 146.08 (109.27–166.22) | 150.74 (101.78–177.46) |
| Both | 95+ years | Rate | 156.51 (119.73–175.09) | 159.72 (112.56–184.64) |

Note: GBD 2021 estimates. Rates are per 100,000 population; native five-year age groups are retained.

**Supplementary Table 5.** Full SEER baseline characteristics by age group.

| Variable | Category | AYA | Middle-aged adults | Older adults | P value |
| --- | --- | --- | --- | --- | --- |
| Total | N | 3588 | 33643 | 52567 |  |
| Sex | Female | 1673 (46.6%) | 11106 (33.0%) | 20559 (39.1%) | P < 0.001 |
| Sex | Male | 1915 (53.4%) | 22537 (67.0%) | 32008 (60.9%) | P < 0.001 |
| Race/ethnicity | Hispanic | 1594 (44.4%) | 8452 (25.1%) | 8830 (16.8%) | P < 0.001 |
| Race/ethnicity | Non-Hispanic Asian or Pacific Islander | 572 (15.9%) | 4857 (14.4%) | 8892 (16.9%) | P < 0.001 |
| Race/ethnicity | Non-Hispanic Black | 408 (11.4%) | 4527 (13.5%) | 5920 (11.3%) | P < 0.001 |
| Race/ethnicity | Non-Hispanic White | 938 (26.1%) | 15331 (45.6%) | 28381 (54.0%) | P < 0.001 |
| Race/ethnicity | Other/Unknown | 76 (2.1%) | 476 (1.4%) | 544 (1.0%) | P < 0.001 |
| Diagnosis period | 2000–2005 | 940 (26.2%) | 8411 (25.0%) | 15046 (28.6%) | P < 0.001 |
| Diagnosis period | 2006–2011 | 908 (25.3%) | 9075 (27.0%) | 13989 (26.6%) | P < 0.001 |
| Diagnosis period | 2012–2017 | 1035 (28.8%) | 9934 (29.5%) | 14121 (26.9%) | P < 0.001 |
| Diagnosis period | 2018–2021 | 705 (19.6%) | 6223 (18.5%) | 9411 (17.9%) | P < 0.001 |
| Primary site | Cardia | 755 (21.0%) | 11531 (34.3%) | 15454 (29.4%) | P < 0.001 |
| Primary site | Non-cardia | 1674 (46.7%) | 14399 (42.8%) | 25826 (49.1%) | P < 0.001 |
| Primary site | Stomach NOS/other | 1159 (32.3%) | 7713 (22.9%) | 11287 (21.5%) | P < 0.001 |
| Histology | Adenocarcinoma, NOS/common variants | 2001 (55.8%) | 24116 (71.7%) | 43259 (82.3%) | P < 0.001 |
| Histology | Mucinous adenocarcinoma | 65 (1.8%) | 687 (2.0%) | 1180 (2.2%) | P < 0.001 |
| Histology | Other retained rare adenocarcinoma variant | 14 (0.4%) | 211 (0.6%) | 316 (0.6%) | P < 0.001 |
| Histology | Signet-ring cell carcinoma | 1508 (42.0%) | 8629 (25.6%) | 7812 (14.9%) | P < 0.001 |
| Grade | Poorly/undifferentiated | 2558 (71.3%) | 20830 (61.9%) | 28538 (54.3%) | P < 0.001 |
| Grade | Unknown | 648 (18.1%) | 5477 (16.3%) | 8200 (15.6%) | P < 0.001 |
| Grade | Well/moderately differentiated | 382 (10.6%) | 7336 (21.8%) | 15829 (30.1%) | P < 0.001 |
| Summary stage | Distant | 2090 (58.2%) | 15350 (45.6%) | 17145 (32.6%) | P < 0.001 |
| Summary stage | Localized | 388 (10.8%) | 5472 (16.3%) | 13287 (25.3%) | P < 0.001 |
| Summary stage | Regional | 967 (27.0%) | 11305 (33.6%) | 16224 (30.9%) | P < 0.001 |
| Summary stage | Unknown | 143 (4.0%) | 1516 (4.5%) | 5911 (11.2%) | P < 0.001 |
| Surgery | No | 2101 (58.6%) | 17650 (52.5%) | 29303 (55.7%) | P < 0.001 |
| Surgery | Unknown | 46 (1.3%) | 368 (1.1%) | 480 (0.9%) | P < 0.001 |
| Surgery | Yes | 1441 (40.2%) | 15625 (46.4%) | 22784 (43.3%) | P < 0.001 |
| Radiotherapy | No/Unknown | 2703 (75.3%) | 23440 (69.7%) | 40720 (77.5%) | P < 0.001 |
| Radiotherapy | Refused | 10 (0.3%) | 262 (0.8%) | 863 (1.6%) | P < 0.001 |
| Radiotherapy | Yes | 875 (24.4%) | 9941 (29.5%) | 10984 (20.9%) | P < 0.001 |
| Chemotherapy | No/Unknown | 937 (26.1%) | 11798 (35.1%) | 32484 (61.8%) | P < 0.001 |
| Chemotherapy | Yes | 2651 (73.9%) | 21845 (64.9%) | 20083 (38.2%) | P < 0.001 |

Note: Values are n (%) unless otherwise stated. AYA, adolescent and young adult. P values were calculated using chi-square tests. Registry-coded treatment variables are descriptive; chemotherapy No/Unknown does not establish confirmed non-treatment.

**Supplementary Table 6.** Competing-risk death composition, cumulative-incidence estimates, and Gray tests.

| Stratum | Age group | Outcome status | n | % |
| --- | --- | --- | --- | --- |
| Primary age groups | AYA | Alive | 803 | 22.4 |
| Primary age groups | AYA | Gastric-cancer-attributable death | 2588 | 72.1 |
| Primary age groups | AYA | Non-cancer death | 104 | 2.9 |
| Primary age groups | AYA | Unknown/missing COD death | 93 | 2.6 |
| Primary age groups | Middle-aged adults | Alive | 6844 | 20.3 |
| Primary age groups | Middle-aged adults | Gastric-cancer-attributable death | 23676 | 70.4 |
| Primary age groups | Middle-aged adults | Non-cancer death | 2494 | 7.4 |
| Primary age groups | Middle-aged adults | Unknown/missing COD death | 629 | 1.9 |
| Primary age groups | Older adults | Alive | 6086 | 11.6 |
| Primary age groups | Older adults | Gastric-cancer-attributable death | 36483 | 69.4 |
| Primary age groups | Older adults | Non-cancer death | 9206 | 17.5 |
| Primary age groups | Older adults | Unknown/missing COD death | 792 | 1.5 |
| Older-adult subgroups | 65–74 | Alive | 3909 | 16.6 |
| Older-adult subgroups | 65–74 | Gastric-cancer-attributable death | 15775 | 67.2 |
| Older-adult subgroups | 65–74 | Non-cancer death | 3404 | 14.5 |
| Older-adult subgroups | 65–74 | Unknown/missing COD death | 404 | 1.7 |
| Older-adult subgroups | 75–84 | Alive | 1878 | 9.3 |
| Older-adult subgroups | 75–84 | Gastric-cancer-attributable death | 14074 | 69.7 |
| Older-adult subgroups | 75–84 | Non-cancer death | 3940 | 19.5 |
| Older-adult subgroups | 75–84 | Unknown/missing COD death | 294 | 1.5 |
| Older-adult subgroups | ≥85 | Alive | 299 | 3.4 |
| Older-adult subgroups | ≥85 | Gastric-cancer-attributable death | 6634 | 74.6 |
| Older-adult subgroups | ≥85 | Non-cancer death | 1862 | 20.9 |
| Older-adult subgroups | ≥85 | Unknown/missing COD death | 94 | 1.1 |

Note: Percentages are calculated within age group. Unknown/missing COD death is distinct from non-cancer death.

**Supplementary Table 7.** Model 1 Cox regression results for overall and cause-specific mortality.

| Endpoint | Variable | Comparison | Reference | HR | 95% CI | P value |
| --- | --- | --- | --- | --- | --- | --- |
| OS | Age group | Middle-aged adults | AYA | 0.94 | 0.90–0.98 | P = 0.002 |
| OS | Age group | Older adults | AYA | 1.25 | 1.20–1.30 | P < 0.001 |
| OS | Sex | Male | Female | 0.99 | 0.97–1.00 | P = 0.096 |
| OS | Race/ethnicity | Non-Hispanic Black | Non-Hispanic White | 1.05 | 1.02–1.07 | P < 0.001 |
| OS | Race/ethnicity | Non-Hispanic Asian or Pacific Islander | Non-Hispanic White | 0.71 | 0.70–0.73 | P < 0.001 |
| OS | Race/ethnicity | Hispanic | Non-Hispanic White | 0.97 | 0.95–0.99 | P = 0.002 |
| OS | Race/ethnicity | Other/Unknown | Non-Hispanic White | 1.05 | 0.98–1.12 | P = 0.153 |
| OS | Diagnosis period | 2006–2011 | 2000–2005 | 0.91 | 0.89–0.93 | P < 0.001 |
| OS | Diagnosis period | 2012–2017 | 2000–2005 | 0.88 | 0.86–0.89 | P < 0.001 |
| OS | Diagnosis period | 2018–2021 | 2000–2005 | 0.82 | 0.80–0.83 | P < 0.001 |
| Cause-specific | Age group | Middle-aged adults | AYA | 0.90 | 0.86–0.94 | P < 0.001 |
| Cause-specific | Age group | Older adults | AYA | 1.03 | 0.99–1.07 | P = 0.135 |
| Cause-specific | Sex | Male | Female | 0.97 | 0.96–0.99 | P = 0.002 |
| Cause-specific | Race/ethnicity | Non-Hispanic Black | Non-Hispanic White | 1.01 | 0.99–1.04 | P = 0.406 |
| Cause-specific | Race/ethnicity | Non-Hispanic Asian or Pacific Islander | Non-Hispanic White | 0.71 | 0.69–0.73 | P < 0.001 |
| Cause-specific | Race/ethnicity | Hispanic | Non-Hispanic White | 0.99 | 0.97–1.01 | P = 0.179 |
| Cause-specific | Race/ethnicity | Other/Unknown | Non-Hispanic White | 1.04 | 0.97–1.12 | P = 0.253 |
| Cause-specific | Diagnosis period | 2006–2011 | 2000–2005 | 0.91 | 0.89–0.93 | P < 0.001 |
| Cause-specific | Diagnosis period | 2012–2017 | 2000–2005 | 0.87 | 0.85–0.89 | P < 0.001 |
| Cause-specific | Diagnosis period | 2018–2021 | 2000–2005 | 0.80 | 0.78–0.82 | P < 0.001 |

Note: Model 1 adjusted for age group, sex, race/ethnicity, and diagnosis period. HR, hazard ratio; CI, confidence interval.

**Supplementary Table 8.** Primary clinicopathologic-adjusted Model 2 Cox regression results for overall and cause-specific mortality.

| Endpoint | Variable | Comparison | Reference | HR | 95% CI | P value |
| --- | --- | --- | --- | --- | --- | --- |
| OS | Age group | Middle-aged adults | AYA | 1.11 | 1.06–1.15 | P < 0.001 |
| OS | Age group | Older adults | AYA | 1.73 | 1.66–1.79 | P < 0.001 |
| OS | Sex | Male | Female | 1.01 | 1.00–1.03 | P = 0.061 |
| OS | Race/ethnicity | Non-Hispanic Black | Non-Hispanic White | 1.06 | 1.04–1.09 | P < 0.001 |
| OS | Race/ethnicity | Non-Hispanic Asian or Pacific Islander | Non-Hispanic White | 0.75 | 0.74–0.77 | P < 0.001 |
| OS | Race/ethnicity | Hispanic | Non-Hispanic White | 0.93 | 0.91–0.95 | P < 0.001 |
| OS | Race/ethnicity | Other/Unknown | Non-Hispanic White | 1.05 | 0.98–1.12 | P = 0.136 |
| OS | Diagnosis period | 2006–2011 | 2000–2005 | 0.88 | 0.86–0.89 | P < 0.001 |
| OS | Diagnosis period | 2012–2017 | 2000–2005 | 0.81 | 0.79–0.82 | P < 0.001 |
| OS | Diagnosis period | 2018–2021 | 2000–2005 | 0.73 | 0.71–0.74 | P < 0.001 |
| OS | Primary site | Cardia | Non-cardia | 1.01 | 0.99–1.03 | P = 0.336 |
| OS | Primary site | Stomach NOS/other | Non-cardia | 1.26 | 1.24–1.29 | P < 0.001 |
| OS | Histologic subtype | Signet-ring cell carcinoma | Adenocarcinoma, NOS/common variants | 1.06 | 1.04–1.08 | P < 0.001 |
| OS | Histologic subtype | Mucinous adenocarcinoma | Adenocarcinoma, NOS/common variants | 0.99 | 0.94–1.03 | P = 0.557 |
| OS | Histologic subtype | Other retained rare adenocarcinoma variant | Adenocarcinoma, NOS/common variants | 1.05 | 0.96–1.16 | P = 0.269 |
| OS | Grade | Poorly/undifferentiated | Well/moderately differentiated | 1.23 | 1.21–1.25 | P < 0.001 |
| OS | Grade | Unknown | Well/moderately differentiated | 1.29 | 1.26–1.32 | P < 0.001 |
| OS | Summary stage | Regional | Localized | 1.55 | 1.51–1.58 | P < 0.001 |
| OS | Summary stage | Distant | Localized | 4.45 | 4.35–4.55 | P < 0.001 |
| OS | Summary stage | Unknown | Localized | 3.04 | 2.95–3.13 | P < 0.001 |
| Cause-specific | Age group | Middle-aged adults | AYA | 1.08 | 1.04–1.13 | P < 0.001 |
| Cause-specific | Age group | Older adults | AYA | 1.52 | 1.46–1.58 | P < 0.001 |
| Cause-specific | Sex | Male | Female | 1.01 | 0.99–1.02 | P = 0.490 |
| Cause-specific | Race/ethnicity | Non-Hispanic Black | Non-Hispanic White | 1.04 | 1.01–1.07 | P = 0.003 |
| Cause-specific | Race/ethnicity | Non-Hispanic Asian or Pacific Islander | Non-Hispanic White | 0.76 | 0.74–0.78 | P < 0.001 |
| Cause-specific | Race/ethnicity | Hispanic | Non-Hispanic White | 0.94 | 0.92–0.96 | P < 0.001 |
| Cause-specific | Race/ethnicity | Other/Unknown | Non-Hispanic White | 1.06 | 0.99–1.14 | P = 0.107 |
| Cause-specific | Diagnosis period | 2006–2011 | 2000–2005 | 0.87 | 0.85–0.89 | P < 0.001 |
| Cause-specific | Diagnosis period | 2012–2017 | 2000–2005 | 0.79 | 0.77–0.81 | P < 0.001 |
| Cause-specific | Diagnosis period | 2018–2021 | 2000–2005 | 0.70 | 0.68–0.72 | P < 0.001 |
| Cause-specific | Primary site | Cardia | Non-cardia | 1.03 | 1.01–1.05 | P = 0.003 |
| Cause-specific | Primary site | Stomach NOS/other | Non-cardia | 1.29 | 1.27–1.32 | P < 0.001 |
| Cause-specific | Histologic subtype | Signet-ring cell carcinoma | Adenocarcinoma, NOS/common variants | 1.09 | 1.06–1.11 | P < 0.001 |
| Cause-specific | Histologic subtype | Mucinous adenocarcinoma | Adenocarcinoma, NOS/common variants | 0.99 | 0.94–1.05 | P = 0.748 |
| Cause-specific | Histologic subtype | Other retained rare adenocarcinoma variant | Adenocarcinoma, NOS/common variants | 1.07 | 0.96–1.18 | P = 0.213 |
| Cause-specific | Grade | Poorly/undifferentiated | Well/moderately differentiated | 1.31 | 1.29–1.34 | P < 0.001 |
| Cause-specific | Grade | Unknown | Well/moderately differentiated | 1.35 | 1.31–1.38 | P < 0.001 |
| Cause-specific | Summary stage | Regional | Localized | 2.04 | 1.98–2.10 | P < 0.001 |
| Cause-specific | Summary stage | Distant | Localized | 6.22 | 6.05–6.39 | P < 0.001 |
| Cause-specific | Summary stage | Unknown | Localized | 3.85 | 3.71–3.99 | P < 0.001 |

Note: Model 2 adjusted for age group, sex, race/ethnicity, diagnosis period, primary site, histologic subtype, grade, and summary stage. HR, hazard ratio; CI, confidence interval.

**Supplementary Table 9.** Exploratory treatment-adjusted Model 3 Cox regression results for overall and cause-specific mortality.

| Endpoint | Variable | Comparison | Reference | HR | 95% CI | P value |
| --- | --- | --- | --- | --- | --- | --- |
| OS | Age group | Middle-aged adults | AYA | 1.08 | 1.04–1.12 | P < 0.001 |
| OS | Age group | Older adults | AYA | 1.46 | 1.40–1.51 | P < 0.001 |
| OS | Sex | Male | Female | 1.05 | 1.04–1.07 | P < 0.001 |
| OS | Race/ethnicity | Non-Hispanic Black | Non-Hispanic White | 1.03 | 1.01–1.06 | P = 0.004 |
| OS | Race/ethnicity | Non-Hispanic Asian or Pacific Islander | Non-Hispanic White | 0.76 | 0.74–0.77 | P < 0.001 |
| OS | Race/ethnicity | Hispanic | Non-Hispanic White | 0.90 | 0.89–0.92 | P < 0.001 |
| OS | Race/ethnicity | Other/Unknown | Non-Hispanic White | 0.95 | 0.89–1.01 | P = 0.101 |
| OS | Diagnosis period | 2006–2011 | 2000–2005 | 0.90 | 0.88–0.91 | P < 0.001 |
| OS | Diagnosis period | 2012–2017 | 2000–2005 | 0.83 | 0.82–0.85 | P < 0.001 |
| OS | Diagnosis period | 2018–2021 | 2000–2005 | 0.76 | 0.74–0.78 | P < 0.001 |
| OS | Primary site | Cardia | Non-cardia | 0.96 | 0.95–0.98 | P < 0.001 |
| OS | Primary site | Stomach NOS/other | Non-cardia | 1.18 | 1.16–1.20 | P < 0.001 |
| OS | Histologic subtype | Signet-ring cell carcinoma | Adenocarcinoma, NOS/common variants | 1.09 | 1.07–1.11 | P < 0.001 |
| OS | Histologic subtype | Mucinous adenocarcinoma | Adenocarcinoma, NOS/common variants | 1.07 | 1.02–1.13 | P = 0.004 |
| OS | Histologic subtype | Other retained rare adenocarcinoma variant | Adenocarcinoma, NOS/common variants | 1.16 | 1.06–1.27 | P = 0.002 |
| OS | Grade | Poorly/undifferentiated | Well/moderately differentiated | 1.28 | 1.26–1.31 | P < 0.001 |
| OS | Grade | Unknown | Well/moderately differentiated | 1.11 | 1.09–1.14 | P < 0.001 |
| OS | Summary stage | Regional | Localized | 2.19 | 2.14–2.24 | P < 0.001 |
| OS | Summary stage | Distant | Localized | 3.62 | 3.53–3.71 | P < 0.001 |
| OS | Summary stage | Unknown | Localized | 1.64 | 1.59–1.69 | P < 0.001 |
| OS | Surgery | Yes | No | 0.33 | 0.33–0.34 | P < 0.001 |
| OS | Surgery | Unknown | No | 0.63 | 0.59–0.68 | P < 0.001 |
| OS | Radiotherapy | Yes | No/Unknown | 1.00 | 0.98–1.02 | P = 0.967 |
| OS | Radiotherapy | Refused | No/Unknown | 0.98 | 0.92–1.04 | P = 0.526 |
| OS | Chemotherapy | Yes | No/Unknown | 0.49 | 0.48–0.50 | P < 0.001 |
| Cause-specific | Age group | Middle-aged adults | AYA | 1.05 | 1.01–1.09 | P = 0.027 |
| Cause-specific | Age group | Older adults | AYA | 1.26 | 1.21–1.31 | P < 0.001 |
| Cause-specific | Sex | Male | Female | 1.04 | 1.02–1.06 | P < 0.001 |
| Cause-specific | Race/ethnicity | Non-Hispanic Black | Non-Hispanic White | 1.01 | 0.98–1.04 | P = 0.450 |
| Cause-specific | Race/ethnicity | Non-Hispanic Asian or Pacific Islander | Non-Hispanic White | 0.77 | 0.75–0.79 | P < 0.001 |
| Cause-specific | Race/ethnicity | Hispanic | Non-Hispanic White | 0.92 | 0.90–0.94 | P < 0.001 |
| Cause-specific | Race/ethnicity | Other/Unknown | Non-Hispanic White | 0.96 | 0.90–1.04 | P = 0.338 |
| Cause-specific | Diagnosis period | 2006–2011 | 2000–2005 | 0.88 | 0.86–0.90 | P < 0.001 |
| Cause-specific | Diagnosis period | 2012–2017 | 2000–2005 | 0.81 | 0.79–0.83 | P < 0.001 |
| Cause-specific | Diagnosis period | 2018–2021 | 2000–2005 | 0.73 | 0.71–0.74 | P < 0.001 |
| Cause-specific | Primary site | Cardia | Non-cardia | 0.97 | 0.95–0.99 | P = 0.011 |
| Cause-specific | Primary site | Stomach NOS/other | Non-cardia | 1.20 | 1.18–1.22 | P < 0.001 |
| Cause-specific | Histologic subtype | Signet-ring cell carcinoma | Adenocarcinoma, NOS/common variants | 1.12 | 1.09–1.14 | P < 0.001 |
| Cause-specific | Histologic subtype | Mucinous adenocarcinoma | Adenocarcinoma, NOS/common variants | 1.08 | 1.03–1.14 | P = 0.004 |
| Cause-specific | Histologic subtype | Other retained rare adenocarcinoma variant | Adenocarcinoma, NOS/common variants | 1.18 | 1.07–1.30 | P = 0.001 |
| Cause-specific | Grade | Poorly/undifferentiated | Well/moderately differentiated | 1.37 | 1.35–1.40 | P < 0.001 |
| Cause-specific | Grade | Unknown | Well/moderately differentiated | 1.16 | 1.13–1.19 | P < 0.001 |
| Cause-specific | Summary stage | Regional | Localized | 2.92 | 2.84–3.01 | P < 0.001 |
| Cause-specific | Summary stage | Distant | Localized | 4.91 | 4.76–5.05 | P < 0.001 |
| Cause-specific | Summary stage | Unknown | Localized | 1.99 | 1.92–2.06 | P < 0.001 |
| Cause-specific | Surgery | Yes | No | 0.31 | 0.30–0.31 | P < 0.001 |
| Cause-specific | Surgery | Unknown | No | 0.66 | 0.61–0.72 | P < 0.001 |
| Cause-specific | Radiotherapy | Yes | No/Unknown | 1.01 | 0.99–1.03 | P = 0.460 |
| Cause-specific | Radiotherapy | Refused | No/Unknown | 1.02 | 0.95–1.09 | P = 0.629 |
| Cause-specific | Chemotherapy | Yes | No/Unknown | 0.48 | 0.47–0.49 | P < 0.001 |

Note: Model 3 additionally included registry-recorded surgery, radiotherapy, and chemotherapy variables and was exploratory. Treatment coefficients should not be interpreted as treatment benefit, effectiveness, or causal effects. Chemotherapy No/Unknown does not establish confirmed non-treatment.

**Supplementary Table 10.** Proportional-hazards diagnostics for primary Model 2.

| Endpoint | Covariate/test | Chi-square | df | P value |
| --- | --- | --- | --- | --- |
| OS | Age group | 44.98 | 2 | P < 0.001 |
| OS | Sex | 38.85 | 1 | P < 0.001 |
| OS | Race/ethnicity | 43.88 | 4 | P < 0.001 |
| OS | Diagnosis period | 71.42 | 3 | P < 0.001 |
| OS | Primary site | 487.72 | 2 | P < 0.001 |
| OS | Histologic subtype | 1.53 | 3 | P = 0.675 |
| OS | Grade | 273.12 | 2 | P < 0.001 |
| OS | Summary stage | 1227.60 | 3 | P < 0.001 |
| OS | Global test | 1933.05 | 20 | P < 0.001 |
| Cause-specific | Age group | 636.26 | 2 | P < 0.001 |
| Cause-specific | Sex | 39.97 | 1 | P < 0.001 |
| Cause-specific | Race/ethnicity | 28.38 | 4 | P < 0.001 |
| Cause-specific | Diagnosis period | 43.96 | 3 | P < 0.001 |
| Cause-specific | Primary site | 533.41 | 2 | P < 0.001 |
| Cause-specific | Histologic subtype | 58.22 | 3 | P < 0.001 |
| Cause-specific | Grade | 139.44 | 2 | P < 0.001 |
| Cause-specific | Summary stage | 1432.31 | 3 | P < 0.001 |
| Cause-specific | Global test | 2613.06 | 20 | P < 0.001 |

Note: Schoenfeld-residual tests were used. Age, the primary exposure of interest, showed evidence of non-proportional hazards for both endpoints; complete covariate and global diagnostics are shown.

**Supplementary Table 11.** Secondary piecewise age Cox estimates for overall and cause-specific mortality.

| Endpoint | Age comparison | Follow-up interval, years | HR | 95% CI | P value | Number at risk at interval start | Events in interval |
| --- | --- | --- | --- | --- | --- | --- | --- |
| OS | Middle-aged adults vs AYA | 0-<1 | 1.09 | 1.04–1.15 | P < 0.001 | 33,572 | 14,827 |
| OS | Older adults vs AYA | 0-<1 | 1.77 | 1.69–1.87 | P < 0.001 | 52,476 | 27,949 |
| OS | Middle-aged adults vs AYA | 1-<3 | 1.04 | 0.97–1.11 | P = 0.325 | 18,190 | 8,172 |
| OS | Older adults vs AYA | 1-<3 | 1.22 | 1.14–1.31 | P < 0.001 | 23,962 | 10,960 |
| OS | Middle-aged adults vs AYA | 3-<5 | 1.09 | 0.93–1.28 | P = 0.294 | 9,176 | 1,783 |
| OS | Older adults vs AYA | 3-<5 | 1.54 | 1.31–1.81 | P < 0.001 | 12,057 | 2,847 |
| OS | Middle-aged adults vs AYA | ≥5 | 1.89 | 1.55–2.29 | P < 0.001 | 6,459 | 1,946 |
| OS | Older adults vs AYA | ≥5 | 5.67 | 4.68–6.88 | P < 0.001 | 8,077 | 4,634 |
| Cause-specific | Middle-aged adults vs AYA | 0-<1 | 1.10 | 1.05–1.16 | P < 0.001 | 32,944 | 13,763 |
| Cause-specific | Older adults vs AYA | 0-<1 | 1.74 | 1.65–1.83 | P < 0.001 | 51,684 | 24,341 |
| Cause-specific | Middle-aged adults vs AYA | 1-<3 | 1.03 | 0.96–1.11 | P = 0.422 | 17,872 | 7,469 |
| Cause-specific | Older adults vs AYA | 1-<3 | 1.14 | 1.06–1.23 | P < 0.001 | 23,541 | 9,006 |
| Cause-specific | Middle-aged adults vs AYA | 3-<5 | 1.01 | 0.85–1.19 | P = 0.941 | 9,056 | 1,466 |
| Cause-specific | Older adults vs AYA | 3-<5 | 1.12 | 0.95–1.33 | P = 0.185 | 11,841 | 1,774 |
| Cause-specific | Middle-aged adults vs AYA | ≥5 | 1.16 | 0.92–1.45 | P = 0.208 | 6,385 | 928 |
| Cause-specific | Older adults vs AYA | ≥5 | 1.90 | 1.52–2.37 | P < 0.001 | 7,935 | 1,290 |

Note: These estimates are secondary interval-averaged associations and do not imply that the proportional-hazards violation was eliminated.

**Supplementary Table 12.** Sensitivity analysis excluding exact-zero-day observations.

| Endpoint | Variable | Comparison | Reference | Primary Model 2 HR | Zero-day-exclusion HR | Relative HR difference |
| --- | --- | --- | --- | --- | --- | --- |
| OS | Age group | Middle-aged adults | AYA | 1.11 | 1.11 | 0.22% |
| OS | Age group | Older adults | AYA | 1.73 | 1.73 | 0.25% |
| OS | Diagnosis period | 2006–2011 | 2000–2005 | 0.88 | 0.88 | 0.03% |
| OS | Diagnosis period | 2012–2017 | 2000–2005 | 0.81 | 0.81 | 0.07% |
| OS | Diagnosis period | 2018–2021 | 2000–2005 | 0.73 | 0.72 | 0.15% |
| OS | Grade | Poorly/undifferentiated | Well/moderately differentiated | 1.23 | 1.23 | 0.01% |
| OS | Grade | Unknown | Well/moderately differentiated | 1.29 | 1.29 | 0.10% |
| OS | Histologic subtype | Mucinous adenocarcinoma | Adenocarcinoma, NOS/common variants | 0.99 | 0.99 | 0.12% |
| OS | Histologic subtype | Other retained rare adenocarcinoma variant | Adenocarcinoma, NOS/common variants | 1.05 | 1.05 | 0.12% |
| OS | Histologic subtype | Signet-ring cell carcinoma | Adenocarcinoma, NOS/common variants | 1.06 | 1.06 | 0.06% |
| OS | Primary site | Cardia | Non-cardia | 1.01 | 1.01 | 0.08% |
| OS | Primary site | Stomach NOS/other | Non-cardia | 1.26 | 1.26 | 0.11% |
| OS | Race/ethnicity | Hispanic | Non-Hispanic White | 0.93 | 0.93 | 0.01% |
| OS | Race/ethnicity | Non-Hispanic Asian or Pacific Islander | Non-Hispanic White | 0.75 | 0.75 | 0.03% |
| OS | Race/ethnicity | Non-Hispanic Black | Non-Hispanic White | 1.06 | 1.06 | 0.00% |
| OS | Race/ethnicity | Other/Unknown | Non-Hispanic White | 1.05 | 1.05 | 0.01% |
| OS | Sex | Male | Female | 1.01 | 1.01 | 0.04% |
| OS | Summary stage | Distant | Localized | 4.45 | 4.46 | 0.16% |
| OS | Summary stage | Regional | Localized | 1.55 | 1.55 | 0.09% |
| OS | Summary stage | Unknown | Localized | 3.04 | 3.03 | 0.15% |
| Cause-specific | Age group | Middle-aged adults | AYA | 1.08 | 1.08 | 0.21% |
| Cause-specific | Age group | Older adults | AYA | 1.52 | 1.52 | 0.20% |
| Cause-specific | Diagnosis period | 2006–2011 | 2000–2005 | 0.87 | 0.87 | 0.04% |
| Cause-specific | Diagnosis period | 2012–2017 | 2000–2005 | 0.79 | 0.79 | 0.05% |
| Cause-specific | Diagnosis period | 2018–2021 | 2000–2005 | 0.70 | 0.70 | 0.13% |
| Cause-specific | Grade | Poorly/undifferentiated | Well/moderately differentiated | 1.31 | 1.31 | 0.03% |
| Cause-specific | Grade | Unknown | Well/moderately differentiated | 1.35 | 1.35 | 0.10% |
| Cause-specific | Histologic subtype | Mucinous adenocarcinoma | Adenocarcinoma, NOS/common variants | 0.99 | 0.99 | 0.11% |
| Cause-specific | Histologic subtype | Other retained rare adenocarcinoma variant | Adenocarcinoma, NOS/common variants | 1.07 | 1.07 | 0.11% |
| Cause-specific | Histologic subtype | Signet-ring cell carcinoma | Adenocarcinoma, NOS/common variants | 1.09 | 1.09 | 0.09% |
| Cause-specific | Primary site | Cardia | Non-cardia | 1.03 | 1.03 | 0.04% |
| Cause-specific | Primary site | Stomach NOS/other | Non-cardia | 1.29 | 1.29 | 0.14% |
| Cause-specific | Race/ethnicity | Hispanic | Non-Hispanic White | 0.94 | 0.94 | 0.01% |
| Cause-specific | Race/ethnicity | Non-Hispanic Asian or Pacific Islander | Non-Hispanic White | 0.76 | 0.76 | 0.00% |
| Cause-specific | Race/ethnicity | Non-Hispanic Black | Non-Hispanic White | 1.04 | 1.04 | 0.03% |
| Cause-specific | Race/ethnicity | Other/Unknown | Non-Hispanic White | 1.06 | 1.06 | 0.03% |
| Cause-specific | Sex | Male | Female | 1.01 | 1.01 | 0.01% |
| Cause-specific | Summary stage | Distant | Localized | 6.22 | 6.22 | 0.13% |
| Cause-specific | Summary stage | Regional | Localized | 2.04 | 2.04 | 0.08% |
| Cause-specific | Summary stage | Unknown | Localized | 3.85 | 3.84 | 0.10% |

Note: Primary analyses retained exact-zero observations at time=0. The sensitivity analysis excluded 214 OS records and 213 cause-specific records. HR, hazard ratio.

**Supplementary Table 13.** Descriptive survival and competing-risk analyses among older adults.

| Variable | Category | 65–74 | 75–84 | ≥85 |
| --- | --- | --- | --- | --- |
| Sex | Male | 15865 (67.5%) | 11968 (59.3%) | 4175 (47.0%) |
| Sex | Female | 7627 (32.5%) | 8218 (40.7%) | 4714 (53.0%) |
| Race/ethnicity | Non-Hispanic Black | 2815 (12.0%) | 2106 (10.4%) | 999 (11.2%) |
| Race/ethnicity | Non-Hispanic White | 12224 (52.0%) | 11153 (55.3%) | 5004 (56.3%) |
| Race/ethnicity | Non-Hispanic Asian or Pacific Islander | 3800 (16.2%) | 3488 (17.3%) | 1604 (18.0%) |
| Race/ethnicity | Hispanic | 4361 (18.6%) | 3240 (16.1%) | 1229 (13.8%) |
| Race/ethnicity | Other/Unknown | 292 (1.2%) | 199 (1.0%) | 53 (0.6%) |
| Diagnosis period | 2000–2005 | 6207 (26.4%) | 6318 (31.3%) | 2521 (28.4%) |
| Diagnosis period | 2006–2011 | 5957 (25.4%) | 5520 (27.3%) | 2512 (28.3%) |
| Diagnosis period | 2012–2017 | 6595 (28.1%) | 5104 (25.3%) | 2422 (27.2%) |
| Diagnosis period | 2018–2021 | 4733 (20.1%) | 3244 (16.1%) | 1434 (16.1%) |
| Primary site | Non-cardia | 10508 (44.7%) | 10279 (50.9%) | 5039 (56.7%) |
| Primary site | Cardia | 8177 (34.8%) | 5494 (27.2%) | 1783 (20.1%) |
| Primary site | Stomach NOS/other | 4807 (20.5%) | 4413 (21.9%) | 2067 (23.3%) |
| Histology | Adenocarcinoma, NOS/common variants | 18836 (80.2%) | 16781 (83.1%) | 7642 (86.0%) |
| Histology | Signet-ring cell carcinoma | 3947 (16.8%) | 2834 (14.0%) | 1031 (11.6%) |
| Histology | Mucinous adenocarcinoma | 536 (2.3%) | 464 (2.3%) | 180 (2.0%) |
| Histology | Other retained rare adenocarcinoma variant | 173 (0.7%) | 107 (0.5%) | 36 (0.4%) |
| Grade | Poorly/undifferentiated | 13282 (56.5%) | 10817 (53.6%) | 4439 (49.9%) |
| Grade | Well/moderately differentiated | 6728 (28.6%) | 6252 (31.0%) | 2849 (32.1%) |
| Grade | Unknown | 3482 (14.8%) | 3117 (15.4%) | 1601 (18.0%) |
| Summary stage | Distant | 8731 (37.2%) | 6396 (31.7%) | 2018 (22.7%) |
| Summary stage | Regional | 8186 (34.8%) | 6160 (30.5%) | 1878 (21.1%) |
| Summary stage | Localized | 5177 (22.0%) | 5378 (26.6%) | 2732 (30.7%) |
| Summary stage | Unknown | 1398 (6.0%) | 2252 (11.2%) | 2261 (25.4%) |
| Surgery | Yes | 11694 (49.8%) | 8764 (43.4%) | 2326 (26.2%) |
| Surgery | No | 11565 (49.2%) | 11265 (55.8%) | 6473 (72.8%) |
| Surgery | Unknown | 233 (1.0%) | 157 (0.8%) | 90 (1.0%) |
| Radiotherapy | No/Unknown | 17039 (72.5%) | 16094 (79.7%) | 7587 (85.4%) |
| Radiotherapy | Yes | 6182 (26.3%) | 3720 (18.4%) | 1082 (12.2%) |
| Radiotherapy | Refused | 271 (1.2%) | 372 (1.8%) | 220 (2.5%) |
| Chemotherapy | No/Unknown | 11064 (47.1%) | 13581 (67.3%) | 7839 (88.2%) |
| Chemotherapy | Yes | 12428 (52.9%) | 6605 (32.7%) | 1050 (11.8%) |
| Vital status | Dead | 19583 (83.4%) | 18308 (90.7%) | 8590 (96.6%) |
| Vital status | Alive | 3909 (16.6%) | 1878 (9.3%) | 299 (3.4%) |
| Death category | Gastric-cancer-attributable death | 15775 (67.2%) | 14074 (69.7%) | 6634 (74.6%) |
| Death category | Non-cancer death | 3404 (14.5%) | 3940 (19.5%) | 1862 (20.9%) |
| Death category | Alive | 3909 (16.6%) | 1878 (9.3%) | 299 (3.4%) |
| Death category | Unknown/missing COD death | 404 (1.7%) | 294 (1.5%) | 94 (1.1%) |

Note: Older-adult groups are 65–74 years, 75–84 years, and ≥85 years.
